# Hierarchic regulation of a metabolic pathway: H-NS, CRP, and SsrB control *myo*-inositol utilization by *Salmonella enterica*

**DOI:** 10.1128/spectrum.02724-23

**Published:** 2023-12-14

**Authors:** Angela Felsl, Dominik Brokatzky, Carsten Kröger, Ralf Heermann, Thilo M. Fuchs

**Affiliations:** 1 Lehrstuhl für Mikrobielle Ökologie, ZIEL-Institute for Food and Health, School of Life Science, Technische Universität München, Freising, Germany; 2 Department of Microbiology, School of Genetics and Microbiology, Moyne Institute of Preventive Medicine, Trinity College Dublin, University of Dublin, Dublin, Ireland; 3 Johannes Gutenberg University Mainz, Institute of Molecular Physiology (imP), Biocenter II, Microbiology and Biotechnology, Mainz, Germany; 4 Friedrich-Loeffler-Institut, Institute of Molecular Pathogenesis, Jena, Germany; Ludwig-Maximilians-Universitat Munchen Pettenkofer Institute, Munich, Germany

**Keywords:** *myo*-inositol, H-NS, CRP, SsrA/SsrB, metabolic regulation, metabolism, *Salmonella enterica*

## Abstract

The metabolic island GEI4417/4436 of *Salmonella enterica* serovar Typhimurium (*S*. Typhimurium) enables the degradation of *myo*-inositol (MI) as the sole carbon and energy source. The island encodes two regulatory factors, the autoregulated repressor IolR that inhibits the transcription of most *iol* genes in the absence of MI, and the activator ReiD that induces the expression of an *iol* gene operon that is not controlled by IolR. In this study, we investigated the putative role of global regulators in the control of MI utilization by *S*. Typhimurium. The histone-like nucleoid structuring protein H-NS is demonstrated here to interact with 16 regions of GEI4417/4436 and to silence the transcriptional activity of the promoters P*_reiD_* and P*_iolE_*, thus controlling the expression of genes initial for MI degradation. The cAMP-binding regulatory protein (CRP) is shown to bind numerous promoters in GEI4417/4436 and is required for the growth of *S*. Typhimurium strain 14028 in a minimal medium with MI. The binding kinetics of H-NS and CRP toward promoters of GEI4417/4436 were quantified by surface resonance spectroscopy, showing that H-NS weakly binds to promoters of initial genes and that CRP has a high affinity to promoters of all genes essential for MI utilization. Furthermore, three promoters of the metabolic island were identified here to belong to the virulon of the two-component system SsrA/SsrB of salmonellae as demonstrated by electrophoretic mobility shift assays and the use of chromosomal luciferase reporter fusions.

**IMPORTANCE:**

The capacity to utilize myo-inositol (MI) as sole carbon and energy source is widespread among bacteria, among them the intestinal pathogen *S*. Typhimurium. This study elucidates the complex and hierarchical regulation that underlies the utilization of MI by *S*. Typhimurium under substrate limitation. A total of seven regulatory factors have been identified so far, allowing the pathogen an environment-dependent, efficient, and fine-tuned regulation of a metabolic property that provides growth advantages in different environments.

## INTRODUCTION

A broad metabolic repertoire of bacterial pathogens is known as a prerequisite for a successful infection as it allows to overcome nutrient limitations encountered in the host (1). The enteropathogen *Salmonella enterica* is known for its metabolic plasticity that creates a robustness toward fluctuations in substrate availability (2, 3). Although the nutritional interface between host and *Salmonella* is quantitatively poor, the large number of potential nutrients accessible by *Salmonella* enables proliferation within distinct compartments of the host (4, 5). Some of these catabolic pathways such as galactitol degradation are considered specific adaptations that increase the fitness of *Salmonella* in competition with the gut microbiota (6 – 8). Two other prominent examples of metabolic adaptation that *S*. Typhimurium benefits from in the intestine are the utilization of ethanolamine, which is a component of phospholipids and of propanediol derived from rhamnose or fucose (9 – 13). Strikingly, the genes encoding both these two cobalamin-dependent pathways are present only in the genomes of the three food-borne pathogens *Listeria monocytogenes*, *Clostridium perfringens*, and *S*. Typhimurium (14).

Another dissimilatory pathway of *S*. Typhimurium is encoded by the genomic island (GEI) STM4417/4436 (Fig. S1A) that confers the capability to utilize *myo*-inositol (MI) as sole carbon and energy source (15). MI is a polyol that is abundantly present in soil, food, and organisms (14, 16). Its phosphorylated form, inositol hexakisphosphate or phytate, serves as the main phosphorus storage molecule in plants and is utilized by livestock in the presence of bacterial phytases. Growth of *S*. Typhimurium on minimal medium (MM) with MI as carbon and energy source displays a bistable phenotype that is characterized by an extraordinarily long lag-phase of approximately 2 days if cells were not yet adapted to MI (17 – 20).

GEI 4417/4436 of *S*. Typhimurium strain 14028 has a length of 22 kb and harbors at least 20 genes of which 15 have been identified to be involved in MIs degradation. The MI degradation cascade requires the enzymes IolG1, IolE, IolD, IolB, IolC, and IolA that finally produce glyceraldehyde-3-phosphate and acetyl-CoA (15). IolT1 was identified as an essential inositol transporter of *S*. Typhimurium in laboratory conditions (21). Genes such as *srfJ*, *iolI1*, *iolG2*, *iolI2*, and *iolH* have not been characterized but predicted functions point to a possible role in MI metabolism (22). The products of three genes, *iolR*, *reiD*, and *rssR*, in GEI4417/4436 regulate MI utilization. The repressor IolR inhibits the transcription from all *iol* promoters, with the exception of P*_iolE_*, during growth in media lacking MI, and *iolR* deletion significantly reduces the long lag-phase and abolishes growth bistability (15, 17). The same effects were obtained by the addition of bicarbonate, an electrolyte that is secreted at large amounts by the proximal duodenum (23, 24), thus synchronizing MI utilization in the *S*. Typhimurium population. The activator ReiD is encoded by an orphan gene, which is negatively controlled by IolR, and stimulates transcription of *iolE* and *iolG1*, the genes encoding the two enzymes that are responsible for the first two steps in MI degradation (25). The sRNA RssR is a regulatory element that stabilizes the *reiD* mRNA by interacting with its 5′-UTR (26). These GEI-intrinsic regulators revealed their functions during *S*. Typhimurium growth *in vitro*, indicating that the concentration of MI in the medium controls *iol* gene expression.

The involvement of non-GEI-intrinsic *Salmonella* master regulators in MI degradation, however, has not been studied in detail. The histone-like nucleoid structuring protein H-NS is a global silencer of AT-rich genes (27), including horizontally acquired gene islands, and interaction of H-NS with several regions within the GEI4417/4436 has been implicated by cDNA microarray analysis and chromatin immunoprecipitation (ChIP) (28, 29). The cyclic AMP receptor protein (CRP) is a global regulator responsible for the control of more than 400 *Salmonella* genes (30). CRP is a good candidate for *iol* gene regulation, as it activates the uptake and degradation of alternative sugars when glucose or other preferred carbon and energy sources are depleted (31). The response regulator SsrB has been identified to induce transcription of *srfJ* that encodes a putative ceramidase of unknown function in MI metabolism (32 – 34). The two-component regulatory system SsrAB activates transcription of the *Salmonella* pathogenicity island 2 (SPI2) that is required for pathogen survival inside macrophage vacuoles. Thus, *srfJ* as part of the SsrAB regulon connects MI metabolism to later stages of intracellular pathogenesis.

In this study, we monitored the effects of the overexpression or lack of H-NS, CRP, and SsrB on the transcription of *iol* genes using luciferase reporter fusions. We purified the regulatory proteins and quantified the interactions between these regulators with potential target promoters by electrophoretic mobility shift assays (EMSAs) and by surface plasmon resonance (SPR) spectroscopy. Mutant growth phenotypes confirmed the role of CRP and SsrA/SsrB in MI utilization by *S. enterica*.

## RESULTS

### H-NS interacts with most *iol* gene promoters

**Fig 1:**
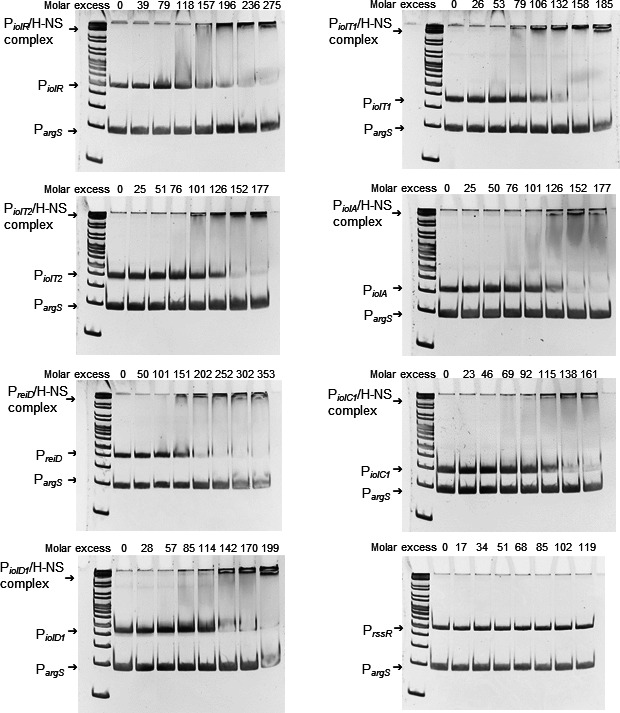
EMSAs with His_6_-H-NS and *iol* gene promoters. EMSAs were performed with increasing amounts of His_6_-H-NS mixed with 100 ng of DNA fragments representing the promoters of *iolR*, *iolT1*, *iolT2*, *iolA, reiD, iolC1*, *iolD1*, or *rssR*. His_6_-H-NS was purified from pBAD-*hns* using *E. coli* strain BL21 (DE3) KB3 that lacks H-NS and the H-NS-like DNA-binding factor StpA. Complex formation between His_6_-H-NS and the respective promoter DNA is indicated. The DNA fragment tested was applied to the first lane in each gel in the absence of H-NS. The promoter of *argS* (P*_argS_*) served as competitive DNA (15). GeneRuler (Fermentas) was used as DNA ladder mix.

Several H-NS binding sites have been identified on GEI4417/4436 using transcriptome comparison and ChIP (28, 29). To corroborate the role of H-NS in controlling *iol* genes, the DNA-binding protein was overproduced in *Escherichia coli* KB3 with plasmid pBAD-*hns*, purified, and tested by EMSAs for binding the promoter regions of the genes *iolR*, *iolT1*, *iolT2*, *iolA*, *reiD, iolC1*, *iolD1*, and *rssR*. Purified H-NS demonstrated preferential binding to each *iol* gene promoter over the promoter of the housekeeping gene *argS* encoding arginyl-tRNA synthase (Fig. 1). A molar excess of approximately 150–200 H-NS molecules toward promoter fragments was sufficient to quantitatively bind the DNA. H-NS did not bind the promoter of *rssR* encoding a small RNA involved in the regulation of MI degradation (26).

In addition to the promoters mentioned above, several other inter- and intragenic H-NS binding sites were predicted to be located on the two gene regions of GEI4417/4436 ranging from *reiD* to *iolG* and *iolD2* to *iolH*, respectively (28, 29). To experimentally verify binding of H-NS to these promoter regions, 10 different DNA fragments of approximately 300 bp carrying potential H-NS binding sites were selected (Fig. S1B). First, two fragments representing the middle (I) and the last third (II) of gene *reiD* were probed against purified H-NS, and the EMSA results shown in Fig. S2 demonstrate that H-NS binds to fragment II but does not interact with fragment I of *reiD*. Then, EMSAs with fragments III to V confirmed the putative H-NS binding regions within the promoter and the coding region of *iolE*. EMSA analysis of the region *iolD2* to *iolH* comprising five genes, which are not essential for MI degradation *in vitro*, demonstrated H-NS binding upstream of *iolG2*, *STM4434*, and *iolH* (fragments VI, VII, and VIII) and also in the middle of *iolI2* (fragment IX) and of fragment X overlapping the last third of *iolI2* and the first third of *iolH*. Fragments II, III, V, VI, VII, and VIII were quantitatively bound by H-NS a molar excess of approximately 40–140, whereas a much lower affinity of the protein was observed for fragments IV, IX, and X.

To investigate whether H-NS binding to the last third of *reiD* influences the transcription of this activator gene, we fused the *luxCDABE* reporter cassette downstream of *reiD*, resulting in strain 14028 *reiD::lux*. We then monitored the luciferase activity of strains 14028 P*_reiD_::lux* and 14028 *reiD::lux* grown in LB medium, in which the *iol* genes are repressed. With 14028 *reiD::lux*, we observed a 15.9-fold lower bioluminescence of 4.6 × 10^2^ RLU/OD_600_ in comparison with that of strain 14028 P*_reiD_::lux* (7.3 × 10^3^ RLU/OD_600_), a finding that points to the relevance of the binding of repressor H-NS to an intragenic region (fragment II) of *reiD*. Taken together, the EMSAs performed here demonstrate that the global gene silencer H-NS interacts with a total of 16 sites within GEI4417/4436, indicating that the regulator H-NS contributes to the negative control of *iol* gene expression.

### Quantification of H-NS-His_6_ binding to *iol* gene promoters

**Fig 2:**
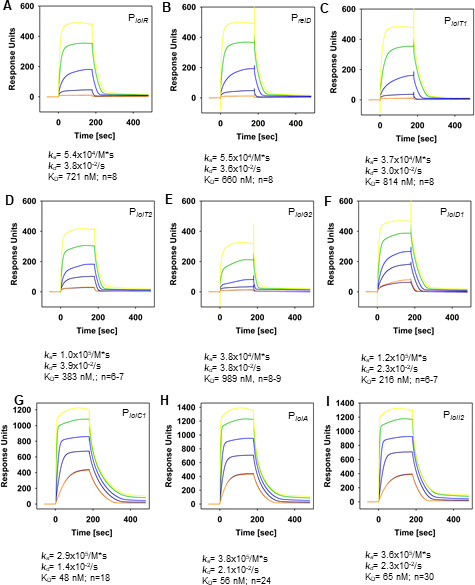
SPR spectroscopy of His_6_-H-NS binding to *iol* promoters. Biotinylated double-stranded DNA fragments comprising the promoter regions of P*_iolR_* (**A**), P*_reiD_* (**B**), P*_iolT1_* (**C**), P*_iolT2_* (**D**), P*_iolG2_* (**E**), P*_iolD1_* (**F**), P*_iolA_* (**G**), P*_iolC1_* (**H**), or P*_iolI2_* (**I**) were captured onto a streptavidin-coated sensor chip, and different concentrations of purified His_6_-H-NS [2 × 35 (violet and orange), 70 nM (dark blue line), 140 nM (blue line), 280 nM (green line), 700 nM (yellow line)] were injected over the chip. Association rates (*k*_a_), dissociation rates (*k*_d_), the overall affinities (*K*_D_), and the binding stoichiometry are depicted below the sensorgrams. All sensorgrams are representatives derived from three independently performed experiments. n.d., not detectable.

To quantify the interaction of H-NS with its target sites on GEI4417/4436 and to determine the corresponding binding kinetics, we performed SPR spectroscopy analyses of H-NS binding to P*_iolR_*, P*_reiD_*, P*_iolT1_*, P*_iolT2_*, P*_iolG2_* (fragment VI in Fig. S1B), P*_iolD1_*, P*_iolA_*, P*_iolC1_*, and P*_iolI2_* (fragment VIII). For that purpose, biotinylated double-stranded DNA fragments comprising the respective promoter regions were captured onto a streptavidin-coated sensor chip, and different concentrations of purified H-NS (0 nM, 2 × 35, 70, 140, 280, and 700 nM) were injected over the chip. The results demonstrated that H-NS interacts with all of promoters tested and that H-NS binding is characterized by fast association (*k*_a_) and fast dissociation (*k*_d_) binding kinetics (Fig. 2). The overall binding affinities varied between 0.2 and 1 µM for the promoters P*_iolR_*, P*_reiD_*, P*_iolT1_*, P*_iolT2_*, P*_iolG2_*, and P*_iolD1_*. However, higher binding affinities of 50–65 nM were observed toward P*_iolC1_*, P*_iolA_*, and P*_iolI2_*. Based on the binding response (RU) and the R_max_, which depends on the molecular weight of H-NS and the respective DNA fragment as well as on the immobilization bulk, we calculated the binding stoichiometry of H-NS toward the DNA fragments containing the respective promoter region. The stoichiometry toward promoters P*_iolR_*, P*_reiD_*, P*_iolT1_*, P*_iolT2_*, P*_iolG2_*, and P*_iolD1_* ranged from *n* = 6 and *n* = 9 and was *n* = 8 in average, suggesting that either more than one binding site for H-NS is located within the DNA fragment, or/and that H-NS binds as higher oligomer. The H-NS binding stoichiometry of the promoters P*_iolA_*, P*_iolC1_*, and P*_iolI2_* was between *n* = 18 and *n* = 30 and, thus, higher compared to the other promoters. These data indicate that the latter three *iol* gene promoters differ from the others not only in higher affinity toward H-NS but also in the number of binding sites and/or in influencing the oligomeric state of the protein. No binding activity was detected toward the control promoter P*_argS_* (data not shown).

### CRP is required for growth with MI

**Fig 3:**
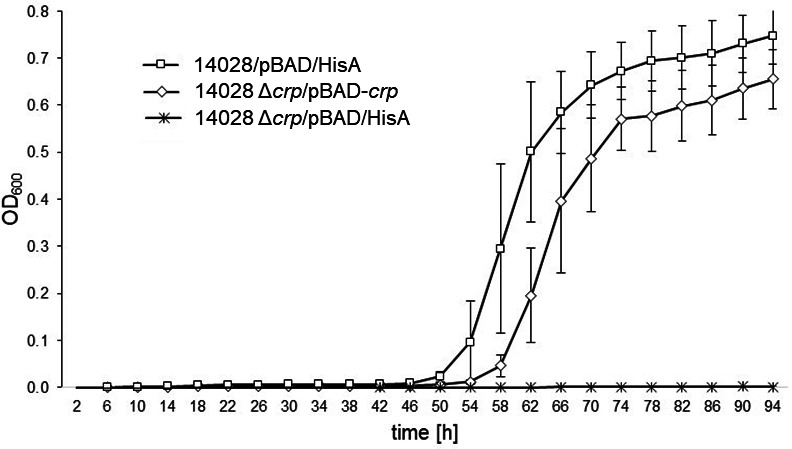
Role of CRP in MI utilization. Growth curves of 14028/pBAD/HisA(Tet^R^), 14028 Δ*crp*/pBAD-*crp*, and14028 Δ*crp*/pBAD/HisA(Tet^R^) in MM with 1% MI in the presence of 1 mM arabinose to induce *crp* expression from the plasmid are shown. Overnight cultures grown in LB were diluted 1:1,000 into MM with MI. The OD_600_ was monitored for 94 h by an Epoch reader; standard deviations of three independently performed experiments are shown.

As glucose is the preferred carbon and energy source for salmonellae, we assumed that the catabolism of MI is under catabolite control. This is supported by the finding that the transcriptional activity of the *iol* promoters in the presence of glucose is below threshold or significantly lower than in the presence of MI (15). To test an involvement of cAMP receptor protein in the regulation of *iol* genes, a mutant with a non-polar deletion of *crp* (14028 Δ*crp*) was constructed. No growth deficiency of this strain compared to the parental strain 14028 was observed in the LB medium (Fig. S3). The Δ*crp* mutant did not grow in MM with MI (Fig. 3). This zero growth phenotype was partially restored to the growth behavior of strain 14028 in the presence of plasmid pBAD-*crp*. We calculated division rates of 0.21 h^−1^ for 14028 and of 0.09 h^−1^ for 14028 Δ*crp*/pBAD-*crp*. As a control, we demonstrated that 14028 Δ*crp* is not able to utilize arabinose for proliferation (Fig. 3).

### CRP binding sites are present in the promoters of all essential *iol* genes but *reiD*

**Fig 4:**
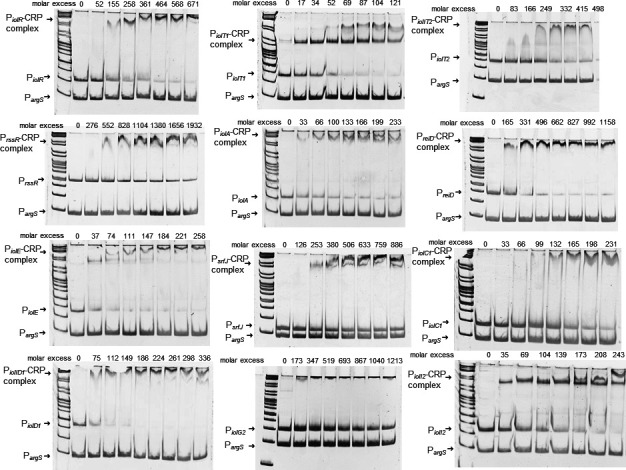
Binding of *iol* gene promoters by CRP-His_6_. For EMSAs, increasing amounts of purified CRP-His_6_ were probed against 100 ng of DNA fragments representing the promoters of *iolR*, *iolT1*, *iolT2*, *rssR*, *iolA, reiD, iolE*, *srfJ*, *iolC1*, *iolD1*, *iolG2*, or *iolG2*. Complex formation between CRP-His_6_ and the respective promoter DNA is indicated. The DNA fragment was applied to the first lane in each gel in the absence of CRP-His_6_.The promoter of *argS* (P*_argS_*) served as competitive DNA. The binding buffer, the gel, and the running buffer contained 20 µM cAMP as co-factor for CRP. Gene ruler (Fermentas) served as DNA ladder mix.

Then, the *iol* promoter regions were analyzed for putative binding by the catabolite gene activator protein CRP. Using a regulatory sequence analysis tool (RSAT) (35) combined with a CRP matrix (36), we identified the consensus sequence TTTTGTGATCTAGATCACAAAA in the regions upstream of *iolT1*, *iolA*, *iolE*, *iolC1*, and *iolD1*, but not within the other *iol* gene promoters (Table S1). To experimentally analyze the putative interaction of CRP with *iol* promoters, CRP was overproduced in *E. coli* TOP10 from plasmid pBAD-*crp* and then used in the presence of cAMP for EMSAs against all promoters located on GEI4417/4436. The promoter region of *argS* served as competitor DNA. The EMSAs shown in Fig. 4 demonstrate the binding of CRP to the promoters of *iolR*, *iolT1*, *iolT2*, *iolA*, *reiD*, *iolE*, *iolC1*, *iolD1*, and *iolI2*. A distinct shift of the P*_iolG2_* fragment was not detected. CRP binding sites are also present upstream of the genes *rssR* and *srfJ*. We did not observe binding of CRP to the fragments upstream of *iolG1* and *iolH*, which were not predicted to carry a promoter fragment and are probably controlled by P*_iolE_* and P*_iolI2_*, respectively (Fig. S4). The molar excess of CRP required for substantial binding of the DNA highly varied between the promoter fragments. Second bands emerging with increasing amounts of CRP, for example, in EMSAs performed with P*_iolT1_* and P*_srfJ_*, point to the binding of a CRP complex to the fragments (37). Taken together, the EMSAs performed here confirmed the predicted binding of the P*_iolT1_*, P*_iolA_*, P*_iolE_*, P*_iolC1_*, and P*_iolD1_* promoters. In addition, we here identified CRP-binding regions upstream of *iolT2*, *rssR*, *reiD*, and *srfJ*. To summarize, the binding of CRP to the promoters of all genes and operons essential for the transport and degradation of *myo*-inositol indicates that CRP contributes to the positive control of *myo*-inositol utilization by *S*. Typhimurium.

### Quantification of CRP-His_6_ binding to *iol* gene promoters

**Fig 5:**
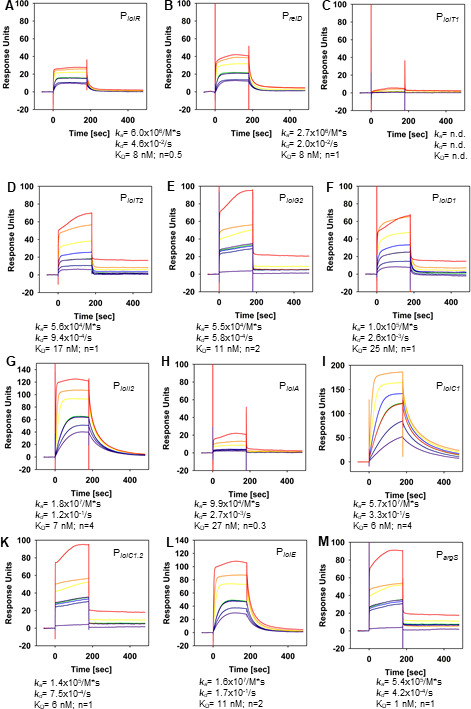
Analysis of CRP/cAMP binding to *iol* promoters via SPR. Biotinylated double-stranded DNA fragments comprising the promoter regions of P*_iolR_* (**A**), P*_reiD_* (**B**), P*_iolT1_* (**C**), P*_iolT2_* (**D**), P*_iolG2_* (**E**), P*_iolD1_* (**F**), P*_iolI2_* (**G**), P*_iolA_* (**H**), P*_iolC1_* (**I**), P*_iolC1.2_* (**K**), P*_iolE_* (**L**), and P*_argS_* (**M**) were captured onto a streptavidin-coated sensor chip, and different concentrations of purified CRP +20 µM cAMP [2.5 nM (purple line), 5 nM (dark blue line), 7.5 nM (green and violet line), 10 nM (blue line), 25 nM (yellow line), 50 nM (orange line), and 100 nM (red line)] were injected over the chip. Association rates (*k*_a_), dissociation rates (*k*_d_), the overall affinities (*K*_D_), and the binding stoichiometry are depicted below the respective sensorgram. All sensorgrams are representatives derived from three independently performed experiments. n.d., not detectable.

To corroborate these data by SPR, different concentrations of purified CRP (0 nM, 2.5 nM, 5 nM, 7.5 nM, 10 nM, 25 nM, 50 nM, and 100 nM) were injected over the chip in the presence and, as control, in the absence of 20 µM cAMP. We determined the binding kinetics of CRP-His_6_ to 12 *iol* gene promoters, namely, P*_iolR_*, P*_reiD_*, P*_iolT1_*, P*_iolT2_*, P*_iolG2_*, P*_iolD1_*, P*_iolI2_*, P*_iolA_*, P*_iolC1_*, P*_iolC1.2_*, P*_iolD1_*, and P*_iolE_*. Promoter fragment P*_iolC1_*, in contrast to P*_iolC1.2_*, carries the consensus sequence mentioned above. In the presence of cAMP, CRP bound to all promoters except P*_iolT1_* (Fig. 5). The most stable and specific binding of CRP/cAMP according to slower dissociation rates and binding saturation was observed for the promoters P*_iolR_*, P*_reiD_*, P*_iolG2_*, P*_iolI2_*, P*_iolC1_*, P*_iolC1.2_*, and P*_iolE_* with binding affinities between 6 and 11 nM. The binding stoichiometry varied between *n* = 0.5 and *n* = 4 and often was *n* = 1, indicating that most promoters carry one CRP binding site. We identified four binding sites, or two binding sites of a CRP dimer, within P*_iolI2_* and P*_iolC1_*. With respect to P*_iolC1.2_* lacking the CRP consensus sequence, we observed a higher dissociation rate and a lower association rate in comparison with P*_iolC1_*. CRP binding toward the control fragment P*_argS_* (Fig. 5M) did not reach saturation under the conditions applied here. Hence, the binding was characterized by fast dissociation rates, indicating a loose and not stable interaction between CRP and P*_argS_* despite an overall affinity of 1 nM.

Without cAMP, CRP binding was detectable only toward P*_iolT2_*, P*_iolG2_*, P*_iolD1_*, and P*_iolC1.2_*. These promoter-CRP interactions were characterized by fast association and fast dissociation rates, resulting in overall affinities between 5 and 100 nM (Fig. S5A through M). Since binding did not reach saturation for all four promoters, we concluded that despite high-affinity constants, the binding of CRP is only loose and not stable. For these four promoters, the sensorgrams were comparable to each other, underlining the loose interaction with a stoichiometry of *n* = 1 or *n* = 2. In summary, our data show that CRP/cAMP interacts with all the different tested *iol* promoters with high affinity, but not with P*_iolT1_*.

### SsrB_c_ contributes to the control of *iolE* and *iolR* transcription

**Fig 6:**
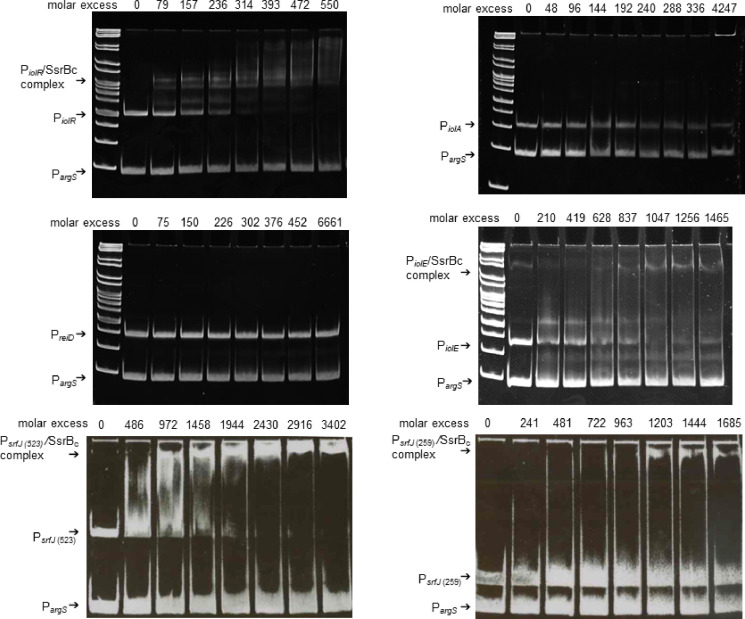
Interaction of His_6_-SsrB_c_ with *iol* gene promoters. EMSAs were performed with increasing amounts of His_6_-SsrB_c_ against 100 ng of DNA fragments representing promoters of *iolR*, *iolA, reiD, and iolE*, and two fragments located upstream of *srfJ*. Complex formation between His_6_-SsrB_c_ and the respective promoter DNA is indicated. The DNA fragment was applied to the first lane in each gel in the absence of SsrB_c_. The promoter of *argS* (P*_argS_*) served as competitor DNA. Gene ruler (Fermentas) served as DNA ladder mix.

We recently demonstrated that SsrB interacts with the promoter of *rssR*, whose product positively regulates inositol degradation by *S*. Typhimurium, and induces *reiD* transcription possibly by binding to its intragenic region but not to P*_reiD_* (26). Footprint analyses with SsrB revealed the consensus binding sequence ACCTGAT that is present in the promoters of *iolR*, *iolA*, *reiD*, and *iolE* (38). To study the interaction of SsrB with GEI4417/4436 regions, we performed EMSAs with the C-terminus of SsrB (SsrB_c_), which had been shown to be constitutively active without conformational activation by SsrA (38). The 3′-fragment of *ssrB* was overproduced from pBAD-*ssrB*c, and purified SsrB_c_ was mixed with fragments representing P*_iolR_*, P*_iolA_*, P*_reiD_*, P*_iolE_*, and two fragments representing 523 and 259 bp upstream of *srfJ*. Specific formation of a SsrB_c_-DNA complex was observed with the promoter fragments P*_iolE_* and P*_iolR_*, but not with P*_iolA_* and P*_reiD_* (Fig. 6). The latter promoter had already been tested previously (26), and the higher maximal molar excess of 6,661 applied here in comparison with 2,638 in our former study confirms a lack of a SsrB_c_-binding site in P*_reiD_*. In comparison with the high binding affinity of SsrB_c_ toward P*_iolR_*, the molar excess required for quantitative binding of P*_iolE_* was more than threefold higher. SsrB_c_ also bound to two fragments located immediately upstream of the *srfJ* start codon, with a higher affinity to the 523 bp fragment as compared to the 259 bp fragment, indicating that this fragment does not harbor all SsrB_c_-binding sites. We conclude that the virulence regulator SsrB interacts with the promoters of the two main regulators of the genes required for MI utilization and also with that of *srfJ*.

**Fig 7:**
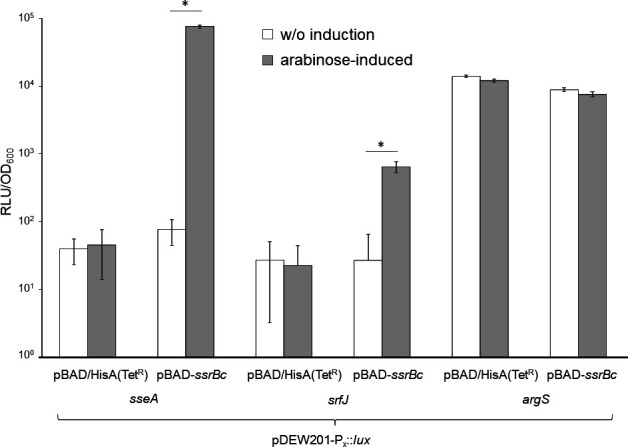
Transcription of *srfJ* is induced by SsrB. Strains *E. coli* TOP10/pDEW201-P*_sseA_::lux*, *E. coli* TOP10/pDEW201-P*_srfJ_::lux*, and *E. coli* TOP10/pDEW201-P*_argS_::lux* were transformed with pBAD-*ssrB*_c_ to allow SsrB_c_ overproduction. Strains transformed with plasmid pBAD/HisA(Tet^R^) instead of pBAD-*ssrB*_c_ served as control. All strains were cultivated in LB medium with tetracycline and ampicillin, and with or without 1 mM arabinose. The maximal transcriptional activities measured as RLUs were normalized to the OD_600_ (RLU/OD_600_). All experiments were performed at least thrice; standard deviations are indicated. Significant differences (*P* < 0.01) are marked by asterisks.

To confirm activation of *srfJ* transcription by SsrB, 252 bp upstream of *srfJ* harboring a putative promoter region was cloned into the *luxCDABE* reporter plasmid pDEW201. As a control, we used pDEW-P*_sseA_::lux* and pDEW-P*_argS_::lux*. The plasmids were transformed into the heterologous host *E. coli* strain TOP10, and the strains were equipped with pBAD/HisA(Tet^R^) and pBAD-*ssrB*_c_, respectively. During growth in LB medium without or with 1 mM arabinose, a more than 10-fold induction of P*_srfJ_* and an approximately 1,000-fold induction of P*_sseA_* was observed (Fig. 7), whereas *ssrB*_c_ overexpression had no significant transcriptional effect on P*_argS_*. Using the same control constructs, we monitored the transcriptional activity of P*_iolE_::lux*, P*_srfJ_::lux*, and P*_iolR_::lux* during the growth of *S*. Typhimurium 14028 lacking *ssrB* in LB and in acidic minimal medium (AMM), which is known to induce the SsrA/SsrB-system. We observed a more than 10-fold induction of P*_srfJ_::lux* in AMM that was abolished upon *ssrB* deletion (Fig. S6). The transcription of the positive control construct P*_sseA_::lux* decreased by several orders of magnitude, whereas that of P*_iolR_::lux* and P*_argS_::lux* was not affected by a deletion of *ssrB* under the conditions applied here. Transcriptional activity of P*_iolE_* was below background in line with the low activity of this promoter (15).

Recently, we demonstrated *via* chromosomal luciferase fusions that overproduction of SsrB_c_ induces the transcription of *reiD* and of the sRNA gene *rssR* (26). To extend the analysis of SsrB-dependent transcription of *iol* genes, chromosomal fusions of the luciferase reporter cassette to *iolR*, P*_iolA_*, P*_reiD_*, *srfJ*, and *sseA* using suicide vector pUTs-*lux*(Cam^R^) were constructed. We chose to use translational *luxCDABE* fusions to the end of the genes *iolR*, *srfJ*, and *sseA* instead of the upstream (promoter) fragments to allow the possible binding of regulatory factors to intragenic regions. The bioluminescence was monitored during growth in LB medium in the presence of pBAD-*ssrB*_c_ without and with arabinose. Strains harboring the empty plasmid pBAD/HisA(Tet^R^) served as a control. As a reference, we used construct 14028 *sseA::lux*/pBAD-*ssrB*_c_ with a fold change FC of 121. The FC values revealed a strong transcriptional induction of *iolR* (FC = 7.5) and *srfJ* (20.3), but no activation of P*_iolA_* (1.9) and P*_reiD_* (2.1) (Table 1). These data confirmed the results of the EMSA experiments. In contrast to P*_reiD_::lux*, the translational fusion *reiD::lux* fusion showed a 155-fold induction in the presence of pBAD-*ssrB*_c_ and arabinose (26). It might be assumed that SsrB binds to the coding region of *reiD* or/and that further regulatory factors compete with SsrB for P*_reiD_* binding under different growth conditions.

**TABLE 1:** Response of selected promoters and genes to the overproduction of SsrB_c_

Strain	Arabinose	RLU/OD_600_	sd (RLU/OD_600_)	Fold change (+/−)
*iolR*::*lux*/pBAD/HisA(Tet^R^)	−	1.7 × 10^4^	1.1 × 10^3^	
	+	3.1 × 10^4^	3.9 × 10^3^	1.8
*iolR*::*lux*/pBAD-*ssrB*c	−	1.5 × 10^4^	360	
	+	1.1 × 10^5^	2.9 × 10^3^	7.5
P*_iolA_*::*lux*	−	7.9 × 10^4^	2.0 × 10^4^	
	+	3.4 × 10^6^	4.7 × 10^5^	43
P*_iolA_*::*lux*/pBAD-*ssrB*c	−	764	204	
	+	1.46 × 10^3^	211	1.9
P*_reiD_*::*lux*/pBAD/HisA(Tet^R^)	−	1.9 × 10^3^	257	
	+	1.6 × 10^3^	160	0.9
P*_reiD_*::*lux*/pBAD-*ssrB*c	−	3.3 × 10^3^	217	
	+	7.0 × 10^3^	209	2.1
*srfJ*::*lux*/pBAD/HisA(Tet^R^)	−	70	30	
	+	110	69	1.6
*srfJ*::*lux*/pBAD-*ssrB*c	−	140	47	
	+	2.9 × 10^3^	310	20.3
*sseA*::*lux*/pBAD/HisA(Tet^R^)	−	2.8 × 10^4^	981	
	+	1.5 × 10^5^	9.6 × 10^3^	5.2
*sseA*::*lux*/pBAD-*ssrB*c	−	4.0 × 10^4^	1.05 × 10^4^	
	+	4.8 × 10^6^	6.0 × 10^5^	121.1

To further analyze the role of the two-component system SsrA/SsrB in MI degradation, we monitored the growth properties of the non-polar deletion mutants 14028Δ*ssrB* and 14028Δ*ssrAB*. Both mutants did not exhibit a significant difference in their growth properties in MI (*P* ≤ 0.004), but their growth curves were characterized by a 2-h shorter lag-phase as compared to parental strain 14028 (Fig. S7). Taken together, we provide experimental evidence that GEI4417/4436 belongs to the regulon of the two-component regulatory system SsrA/SsrB that slightly activates MI degradation *in vitro* upon interaction with the promoter of IolR. Moreover, our data suggest that stimulation of *srfJ* transcription by SsrA/SsrB plays a yet unknown *in vivo* role in MI utilization.

## DISCUSSION

**Fig 8:**
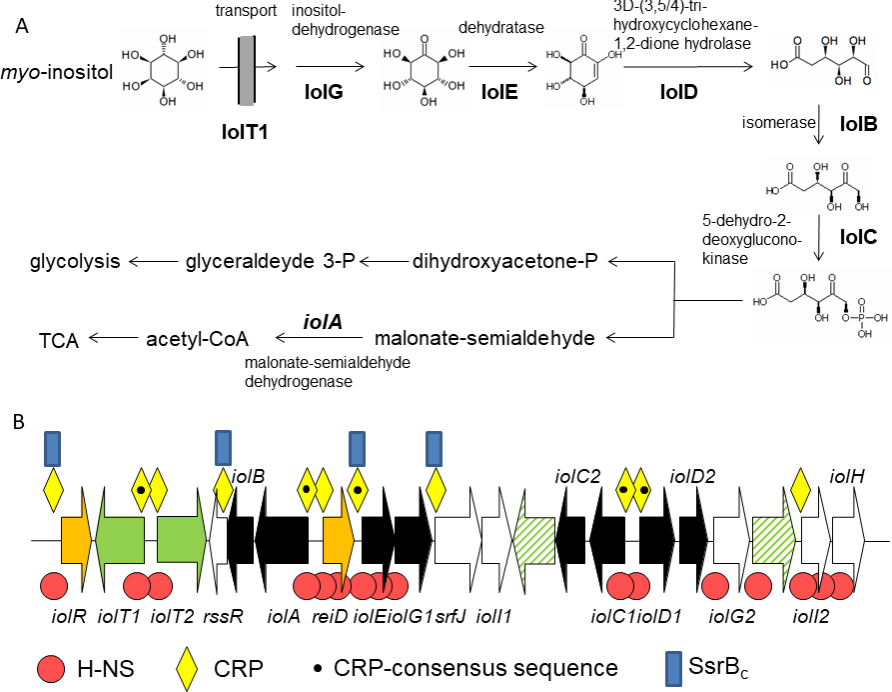
Pathway scheme and regulation. (**A**) MI degradation pathway in *S*. Typhimurium. Names and functions of enzyme involved in the degradation of MI are depicted. (**B**) Binding regions of H-NS, SsrB_c_, and CRP within GEI STM4417/4436. The scheme shows all promoter and intragenic regions to which binding of the three regulators was observed in this study. A dot in the CRP symbol indicates the presence of the CRP-binding consensus sequence. Color code: orange, regulatory genes; green, transporter genes of known or unknown (dashed) function; black, genes essential for MI degradation; white, yet uncharacterized genes not essential for this metabolism.

MI degradation is a widespread, yet neglected metabolic capability of approximately 27% of all bacterial species (22), pointing to its relevance under environmental and *in vivo* conditions. In *S*. Typhimurium, the pathway is tightly repressed by IolR (39), whereas ReiD (25), the small RNA RssR (26), and the catabolic intermediate DKGP positively regulate the enzymatic activities involved in MI utilization. In addition to these intrinsic factors with respect to GEI STM4417/4436, we here identified master regulators that negatively (H-NS) or positively (SsrB and Crp) interact with the transcription of *iol* genes (Fig. 8). H-NS binds broadly to a huge number of sites within GEI4417/4436, thus contributing to *iol* gene silencing in the absence of MI. In contrast, MI degradation is stimulated *in vitro* and in the absence of glucose by CRP that interacts with the promoters of all genes essential for this pathway including the activator gene *reiD*. Noteworthy, the pivotal role of ReiD (and IolE/IolG1) is illustrated by the fact that their production is controlled by all regulators investigated here. Once the first MI molecules are taken up and metabolized, the pathway activation is further activated by the intermediate DKGP and fine-regulated by the small RNA RssR. Given that SsrA/SsrB controls virulence factors, we assume that this TCS particularly triggers the MI metabolism during *S*. Typhimurium infection.

H-NS mediates the silencing of laterally acquired genes in bacteria. Unless the H-NS-bound sequences are integrated into preexisting regulatory networks (28, 29), H-NS removal often has deleterious consequences for the bacterial fitness. An *hns* mutant was not tested here as mutations in *hns* are highly pleiotropic, and loss of *hns* in *Salmonella* results in severe growth effects (27, 40). This is in line with our finding that a Δ*iolR* mutant suffers from a growth disadvantage in rich medium due to untimely *iol* gene expression (39). The H-NS-binding stoichiometries observed here point to distinct oligomeric states of the protein with respect to the individual promoters, a finding that is in line with the literature on H-NS oligomerization (41). Remarkably, we found an accumulation of H-NS binding sites within the promoter and codon regions of genes *reiD* and *iolE* whose products initiate MI utilization and, thus, are pivotal for this pathway (25). The interaction of H-NS with at least 16 sites on GEI4417/4436 (Fig. 8), which was probably acquired by horizontal gene transfer, suggests that MI is not a common substrate of *S*. Typhimurium and that this pathogen prefers other carbon sources.

In our study, we identified CRP as a further factor playing a role in the complex regulation of MI utilization by *S*. Typhimurium. CRP binds to 11 sites located in GEI4417/4436, thus activating the genes involved in regulation, transport, and degradation of MI. In *B. subtilis*, even two distinct catabolite-responsive elements were found to be involved in catabolite repression of the MI degradation operon (42). CRP binding to its specific site on the target DNA is expected to generate a bandshift representative of one CRP homodimer (43) and a DNA fragment. Depending on the size of DNA molecule and location of the CRP site in the DNA molecule, multiple CRP molecules may bind the DNA molecule at additional sites as protein concentrations increase, resulting in super-shifts. We confirmed this assumption as CRP-DNA binding shows non-specific super-shifts at higher protein concentrations as visible in most EMSAs of Fig. 4.

The response regulator SsrB is known to activate several SPI-2 promoters and to counter silencing by H-NS (44, 45). SsrB activates a global regulon, and at least 10 genes outside SPI-2 are SsrB regulated within epithelial and macrophage cells, among them *srfJ* (33) located within GEI4417/4436. The gene *srfJ* (STM4426) has earlier been shown to be induced within host cells by the two component-system (TCS) SsrA(SpiR)/SsrB, which controls the expression of *Salmonella* pathogenicity island- (SPI-) two genes (32 – 34) and is negatively controlled by IolR (15, 46). For binding of P*_iolR_* by SsrB_c_, we demonstrate a specific bandshift that transitions to a supershift at higher protein concentrations. The binding of SsrB to the promoter of *iolR* is intriguing because it fits with a network logic that effects a cross-talk between the repressor IolR and SsrB, thus threading the activator into control of GEI4417/4436.

A binding of SsrB_c_ to the promoter of *srfJ* was not observed by ChIP-on-chip and cDNA hybridization analyses (47). We showed by EMSA that SsrB_c_, indeed, interacts with P*_srfJ_*, indicating that *srfJ* belongs to the SsrA/SsrB virulon (34). SrfJ is a putative glycosyl ceramidase or glycohydrolase (48) that, however, has not directly been linked with MI metabolism so far. Deletion of *srfJ* results in a slight attenuation with respect to the systemic virulence in mice (49). This is in line with our observations that the transcription of *srfJ* is stimulated under acidic conditions, pointing to an *in vivo* role of this enzyme that may fuel the MI metabolism by cleaving MI or related substrates from host molecules. Previous *in vivo* screening experiments, indeed, support the assumption that MI catabolism plays a role in *S*. Typhimurium virulence in mice and pigs (50, 51). A so-called TraDIS-based approach systematically tested a transposon mutant library in chicken, pigs, calves, and mice and revealed a strong attenuation of *iolR*, STM4423, *iolG1*, *iolG2*, STM4434, and *iolI2* mutants in the enteritis models of these animals (52).

### Conclusion

The MI metabolism discussed here is an example of a pathway that confers growth advantages to *S*. Typhimurium under ambient conditions and evolved to a capacity supporting infection of eukaryotic hosts. Our study sheds further light on the complex and hierarchical regulation of this pathway that allows this pathogen to effectively exploit MI as a carbon and energy source. MI utilization by *S*. Typhimurium is also an intriguing example of how bacteria finely tune their gene expression to adapt to environments with shifting nutrient constraints. An improved understanding of this regulatory system is crucial to further elucidate metabolic niche occupation by *S*. Typhimurium.

## MATERIALS AND METHODS

### Bacterial strains, plasmids, and growth conditions

Bacterial strains and plasmids used in this study are listed in Table 2. S. Typhimurium and *E. coli* cultures were grown in Luria-Bertani (LB) medium (10 g/L tryptone, 5 g/L yeast extract, 5 g/L NaCl), in the acidic minimal medium [AAM; 5 mM KCl, 7.5 mM (NH_4_)_2_SO_4_, 0.5 mM K_2_SO_4_, 0.337 mM KH_2_PO_4_, 38 mM glycerin, 0.1% (wt/vol) casamino acids, 80 mM MES, and 8 µM MgCl_2_] (53), in minimal medium [MM; M9 medium supplemented with 2 mM MgSO_4_, 0.1 mM CaCl_2_, and 55.5 mM (1%, wt/vol) MI or 27.8 mM (0.5%, wt/vol) glucose]. For plasmid maintenance, the media were supplemented with the following antibiotics: ampicillin (150 µg/mL), kanamycin (50 µg/mL), tetracycline (12 µg/mL), or chloramphenicol (20 µg/mL). For solid media, 1.5% agar (wt/vol) was added. If appropriate, strains were cultivated in the presence of 5% CO_2_ in a cell culture incubator, or in the presence of 0.1% (11.9 mM) bicarbonate. For all growth and promoter probe experiments, bacterial strains were grown overnight at 37°C and diluted 1:1,000 in liquid growth medium, or streaked on agar plates. One millimolar of (0.015% wt/vol) L(+) arabinose was used to stimulate the expression of genes cloned in pBAD/His(Tet^R^). Growth curves were obtained from bacterial cultures in 96-well microtiter plates incubated at 37°C by an automatic reader (Epoch2T; BioTek, Bad Friedrichshall, Germany). The OD_600_ was measured in time intervals as indicated. Division rates were calculated as *ν* = *n*/*t* = (lg*N−*lg*N*_0_)/[lg2 (*t−t*_0_)] with *N* as the cell number at the chosen timepoints.

**TABLE 2:** Strains and plasmids used in this study

Bacterial strain or plasmid	Description and relevant features	Source or literature
*E. coli* strains		
DH5α	*deoR*, *endA1, gyrA96*, *hsdR17*(r_k_-m_k+_), *recA1*, *relA1*, s*upE44*, *λthi-1*, ∆(*lacZYA-argFV169*)	(54)
SM10 λ*pir*	*lacY tonA recA* Mu_c_ + *thi thr leu supE* RP4-2-Tc::Mu Km^R^λ*pir*	(55)
BL21 (DE3)	*F^-^, omp*T*, hsd*S_B_ (*r_B_^−^m_B_^−^), gal, lon, dcm, rne1*31, λ (DE3 [*lacI lacUV*5-T7 gene 1 *ind1 sam7 nin5*]	(56)
BL21 (DE3) KB3	BL21 (DE3) KB3, ∆*stpA*, ∆*hns*	(57)
TOP10	*F^−^, mcrA, D(mcr-hsdRMS-mcrBC)Φ80lacZΔM15 ΔlacX74 recA1 araD139 Δ(ara-leu)7679, galU, galK, rpsL (Str^r^), endA1, nupG*	Invitrogen, Karlsruhe, Germany
XL-1 Blue	*endA1, gyrA96*, *hsdR17*(r_k_-m_k+_), *recA1*, *relA1*, s*upE44*, *λthi-1*, *lac* [F´ *proAB lacI^q^ ZΔM15* Tn*10* (Tet^R^)]	Agilent, Böblingen, Germany
*S*. Typhimurium strains		
14028	*S*. Typhimurium strain ATCC14028, spontaneous Str^R^ mutant	(15)
14028 ∆*ssrAB*	In-frame *ssrA*/*ssrB* deletion mutant	(58)
14028 ∆*ssrB*	In-frame *ssrB* deletion mutant	(58)
14028 ∆*crp*	In-frame *crp* deletion mutant	This study
14028 P*_iolA_*::*lux*	Chromosomal fusion of the luciferase reporter to the *iolA* promoter	This study
14028 P*_reiD_*::*lux*	Chromosomal fusion of the luciferase reporter to the *iolE* promoter	(25)
14028 *reiD*::*lux*	Chromosomal fusion of the luciferase reporter downstream of *iolE*	(26)
14028 P*_argS_*::*lux*	Chromosomal fusion of the luciferase reporter to the *argS* promoter	(26)
14028 *iolR*::*lux*	Chromosomal fusion of the luciferase reporter downstream of *iolR*	(25)
14028 *srfJ*::*lux*	Chromosomal fusion of the luciferase reporter downstream of *srfJ*	This study
14028 *sseA*::*lux*	Chromosomal fusion of the luciferase reporter downstream of *sseA*	(25)
Plasmids		
pKD4	*pir*-dependent, FRT sites; Kan^R^	CGSC,Yale (59)
pKD46	Lambda-Red helper plasmid; Amp^R^	CGSC,Yale (59)
pCP20	FLP recombinase plasmid; Cm^R^ Amp^R^	CGSC,Yale (59)
pUTs-*lux*(Cm^R^)	Suicide plasmid derived from pUT mini-Tn*5 luxCDABE* Km2 (60) without transposon, and with Kan^R^ substituted by Cm^R^	(61)
pUTs-P*_iolA_*::*lux*	pUTs-*lux*(Cm^R^) plus 500 bp iolA promoter fragment fused with *lux via Sac*I/*Kpn*I	This study
pUTs-*srfJ*::*lux*	pUTs-*lux*(Cm^R^) plus last 500 bp of *srfJ* fused with *lux via Sac*I/*Kpn*I	This study
pDEW201	Promoter probe vector, *luxCDABE*, Amp^R^	(62)
pDEW201-P*_sseA_*::*lux*	pDEW201 with 400 bp upstream of *sseA*	(25)
pDEW201-P*_argS_*::*lux*	pDEW201 with 244 bp upstream of *argS*	(15)
pDEW201-P*_iolR_*::*lux*	pDEW201 with 299 bp upstream of *iolR*	(15)
pDEW201-P*_iolE_*::*lux*	pDEW201 with 321 bp upstream of *iolR*	(15)
pDEW201-P*_srfJ_*::*lux*	pDEW201 with 252 bp upstream of *srfJ* cloned *via Eco*RI/*Kpn*I	This study
pBAD/HisA(Tet^R^)	Derivative of pBAD/HisA (Invitrogen, Carlsbad, USA) with Tet^R^ instead of Amp^R^	(61)
pBAD-*hns*	Gene *hns* of *S*. Typhimurium strain 14028 cloned into pBAD/HisA(Tet^R^) with C-terminal His_6_-tag *via Sac*I and *Eco*RI	This study
pBAD-*ssrB*_c_	3′-end of *ssrB* of *S*. Typhimurium encoding the C-terminus cloned into pBAD/HisA(Tet^R^) with N-terminal His_6_-tag *via Sac*I and *Kpn*I	(26)
pBAD-*crp*	Gene *crp* of *S*. Typhimurium strain 14028 cloned into pBAD/HisA(Tet^R^) with C-terminal His_6_-tag *via Sac*I and *Eco*RI	This study

### Standard procedures

DNA manipulations and isolation of chromosomal or plasmid DNA were performed according to standard protocols (63), and following the manufacturers’ instructions. Plasmid DNA was transformed *via* electroporation by using a Bio-Rad Gene pulser II as recommended by the manufacturer and as described previously (64). Polymerase chain reactions (PCRs) were carried out with Taq polymerase (Fermentas, St. Leon-Rot, Germany). As template for PCR, chromosomal DNA, plasmid DNA, or cells from a single colony were used. Oligonucleotides synthesized for PCRs are listed in Table S1. *S*. Typhimurium gene numbers refer to the LT2 annotation (NC 003197). The Basic Local Alignment Search Tool (BLAST) on the NCBI homepage was used to determine the distribution of *S*. Typhimurium ORFs in the genomes of Gram-negative species. In-frame deletion mutants of *ssrB* and *ssrAB* were constructed by the one-step method based on the phage λ red recombinase as described (59, 65).

### Cloning of promoter fusion to *luxCDABE*

To chromosomally fuse the *luxCDABE* cassette with promoters or the coding sequences of *iol* genes (Table 2), regions spanning 500 bp upstream of the start codon or the stop codon were amplified from chromosomal DNA of *S*. Typhimurium 14028 by PCR using the primers listed in Table S2. The fragments were then cloned upstream of the promoterless *luxCDABE* genes *via Sac*I and *Kpn*I into the multiple cloning site of the suicide vector pUTs-*lux*(Cm^R^). After transformation into *E. coli* SM10 cells, plasmids containing the correct transcriptional *lux*-fusion were isolated and verified by PCR and sequencing. The constructs were then transferred into 14028 by conjugation, and exconjugants were selected and validated by PCR. Enzymes (Fermentas) used are listed in Table 2 and Table S2. To use the reporter plasmid pDEW201 harboring *luxCDABE*, 251 bp comprising the promoter of *srfJ* were cloned *via Eco*RI and *Kpn*I as described (15).

### Quantification of transcriptional activities

Bioluminescence measurements were performed as described recently (15). For growth in MM with MI, bacterial cells were grown at 37°C in 15 mL tubes without agitation. At appropriate time points, 200 µL of each sample was transferred to a 96-well plate, and the cultures were incubated until reaching the stationary phase. OD_600_ and RLU values were recorded in a Wallac VICTOR^3^ 1420 multilabel counter (Perkin Elmer Life Sciences, Turku, Finland). For measurements in LB medium, cells were grown overnight at 37°C and diluted 1:1,000 in LB medium. Samples of 200 µL were then analyzed in a 96-well plate incubated at 37°C under shaking. Three biologically independent experiments were performed for each data point.

### Purification of CRP, SsrB_c_, and H-NS

CRP and SsrB_c_ were overproduced and purified as described recently (7). His_6_-H-NS was overproduced from pBAD-*hns* in *E. coli* BL21 (DE3) KB3 to avoid host-derived H-NS-like contamination. Purification was done using the Ni-NTA Fast Start Kit (Qiagen, Hilden, Germany). Briefly, an overnight culture of *E. coli* was diluted 1:100 into 400 mL LB medium and incubated at 37°C and 180 rpm. After 3 h, the expression of *hns* was induced by adding 1 mM arabinose. After incubation for further 4 h, the cells were harvested and the pellet was resuspended in 4 mL of native lysis buffer. The cells were lysed using ultrasonification (Sonopuls UW2200, Bandelin, Berlin), and the cell debris was removed by centrifugation at 4°C (20 min, 1.6 × 10^4^ g) and filtration via Millex-GV (Merck, Cork, Ireland). The column was then washed and eluted according to the manufacturer’s protocol. The protein concentration was determined using RotiQuant solution (Carl Roth GmbH, Karlsruhe, Germany) based on the method of Bradford (66). The purity of eluted fractions was analysed by separation on a 12.5% SDS polyacrylamide gel and Western blot according to (61) revealing a ~20 kD protein. The protein concentrations were determined using RotiQuant solution (Carl Roth GmbH, Karlsruhe, Germany) based on the method of Bradford (66), and the purity of eluted fractions was analyzed by separation on a 15% SDS polyacrylamide gel revealing a ~34 kD protein.

### Electrophoretic mobility shift assays

For EMSAs, putative binding sequences and competitor DNA P*_argS_* were amplified (for oligonucleotides, see Table S2) and 100 ng of DNA was then mixed with increasing amounts of purified CRP-His_6_, His_6_-SsrB_c_, or His_6_-H-NS in 1 × Tris/borate/EDTA buffer (TBE) with a total volume of 20 µL. After incubation for 45 min at room temperature, the samples were loaded with 4 µL of 6 × loading dye (Fermentas) on a 12% native polyacrylamide gel prepared in 1 × Tris/borate/EDTA buffer and separated at 120 V for 2 h in the same buffer. GeneRuler (Thermo Fisher Scientific, Braunschweig, Germany) was used as DNA ladder mix. DNA was then stained in ethidium bromide solution and visualized by UV irradiation. In case of CRP, the binding buffer, the gel and the running buffer contained 20 µM cAMP as co-factor to enhance DNA-binding activity (37).

### SPR spectroscopy

SPR spectroscopy was performed in a Biacore T200 (Cytiva) using Xantec SAD500-L carboxymethyl dextran sensor chips pre-coated with streptavidin (XanTec Bioanalytics GmbH, Düsseldorf, Germany). All experiments were carried out at a constant temperature of 25°C using HBS-EP + buffer [10 mM HEPES pH 7.4; 150 mM NaCl; 3 mM EDTA; 0.05% (vol/vol) detergent P20]. The chips were equilibrated by three injections using 1 M NaCl/50 mM NaOH at a flow rate of 10 µL/min. Then, 10 nM of a double-stranded biotinylated DNA fragment was injected using a contact time of 300 s and a flow rate of 10 µL/min. As a final activation step, 1 M NaCl/50 mM NaOH/50% (vol/vol) isopropanol was injected. Then, the biotinylated DNA at a concentration of about 10 nmol/L in HBS-EP + buffer (+0.5 M NaCl) was immobilized at a density of each approximately 300–500 response units (RU). Flow cell 1 on each chip was left blank and used to obtain blank sensorgrams for subtraction of bulk refractive index background. Different concentrations of HRP or CRP, respectively, were injected over the chip using an association time of 180 s and a dissociation of 300 s. The interaction of CRP to the different promoter regions was tested without and with 20 µM cAMP. After each cycle, the surface was regenerated by injection of 2.5 M NaCl for 60 s at 30 µL/min flow rate. Sensorgrams were recorded using the Biacore T200 Control software 3.2 and analyzed using Biacore T200 Evaluation software 3.2 (Cytiva). The referenced sensorgrams were normalized to a baseline of 0. Peaks in the sensorgrams at the beginning and the end of the injection emerged from the runtime difference between the flow cells of each chip.

Calibration-free concentration analysis (CFCA) was performed using a 5 µM solution of purified H-NS or 1 µM CRP, respectively, calculated from Lowry-based protein determination, which was stepwise diluted 1:2, 1:5, 1:10, and 1:20. Each protein dilution was two-time injected, one at 5 µL min^−1^ as well as 100 µL min^−1^ flow rate. On the active flow cell P*_iolA_,* P*_iolC1_,* and P*_iol2_*-DNA was used for H-NS-binding, and P*_iolE_,* P*_iolA_,* and P*_iol2_*-DNA for CRP. CFCA basically relies on mass transport, which is a diffusion phenomenon that describes the movement of molecules between the solution and the surface. The CFCA, therefore, relies on the measurement of the observed binding rate during sample injection under partially or complete mass transport limited conditions. Overall, the initial binding rate (d*R*/d*t*) is measured at two different flow rates dependent on the diffusion constant of the protein. The diffusion coefficients of H-NS and CRP were calculated using the Biacore diffusion constant calculator and converter webtool (https://www.cytivalifesciences.com/en/us/solutions/protein-research/products-and-technologies/diffusion-coefficient-calculator), whereby a globular shape of the protein was assumed. The diffusion coefficient of HRP was determined as *D* = 1.225 × 10^−10^ m^2^/s, and of CRP as *D* = 6.902 × 10^−11^ m^2^/s. The initial rates of those dilutions that differed in a factor of at least 1.5 were considered for the calculation of the “active” concentration, which was determined as 7 × 10^−7^ M (14% of the total protein concentration) for H-NS and 1.2 × 10^−7^ M (10% of the total protein concentration) for CRP. The “active” protein concentration was then used for calculation of the binding kinetic constants. Binding stoichiometry (*n*) was calculated. For that purpose, first *R*_max_ was calculated [*R*_max_ = (MW_analyte_/MW_ligand_) ×RU_immobilized ligand_], calculating the maximal binding response for a 1:1 interaction. Binding stoichiometry was then calculated (*n* = RU_max_/R_max_).

## SUPPLEMENTAL MATERIAL

Supplemental figure legends spectrum.02724-23-s0001.pdf Legends of Fig. S1 to S7 and Tables S1 and S2.

Fig. S1 to S7 spectrum.02724-23-s0002.pdf Supplementary figures.

Table S1 spectrum.02724-23-s0003.doc MI-island CRP sites.

Table S2 spectrum.02724-23-s0004.docx Oligonucleotides used in this study.

ASM does not own the copyrights to Supplemental Material that may be linked to, or accessed through, an article. The authors have granted ASM a non-exclusive, world-wide license to publish the Supplemental Material files. Please contact the corresponding author directly for reuse.
