# Supplementary material for: Hierarchic regulation of a metabolic pathway: H-NS, CRP, and SsrB control myo-inositol utilization by Salmonella enterica
Source: Microbiol Spectr. 2023 Dec 14;12(1):e02724-23. doi: 10.1128/spectrum.02724-23 (PMC10783015; doi:10.1128/spectrum.02724-23)
Supplement: Supplemental figure legends — Legends of Fig. S1 to S7 and Tables S1 and S2. [file spectrum.02724-23-s0001.pdf]

**Fig. S1. (A)** Genetic organization of GEI4417/4436. Genes essential for MI degradation are depicted in black, regulatory genes in grey, and yet uncharacterized genes in white. Transporter genes are dashed. **(B)** Fragments I-X used in Fig. S2 to probe $His_6$-H-NS binding by EMSAs (I-X). Numbers at vertical lines mark the putative H-NS binding sites within GEI4417/4436 identified recently [29]. Numbers in brackets indicate the first and the last nucleotide of genes and fragments.

**Fig. S2.** $His_6$-H-NS binding of DNA fragments localized in the region *reiD* to *iolG1* and *iolD2* to *iolH*, respectively. EMSAs were performed with increasing amounts of $His_6$-H-NS mixed with 100 ng of DNA fragments representing the fragments I-X as depicted in Fig. S1. Complex formation between $His_6$-H-NS and the respective promoter DNA is indicated. The DNA fragment tested was applied to the first lane in each gel in the absence of H-NS. The promoter of *argS* ($P_{argS}$) served as competitive DNA. GeneRuler (Fermentas) was used as DNA ladder mix.

**Fig. S3.** Growth curves of *S.* Typhimurium strains 14028, 14028 Δ*crp*, and 14028 Δ*crp*/pBAD-*crp* in LB. Monitoring of the $OD_{600}$ was done for 25 hours by an Epoch reader. Mean values of three independently performed experiments are shown.

**Fig. S4.** EMSAs with CRP-$His_6$ in the presence of cAMP against fragments representing the promoters of *iolG1* and *iolH*. The DNA fragment was applied to the first lane in each gel in the absence of CRP-$His_6$. Complex formation was not observed. The promoter of *argS* ($P_{argS}$) served as competitive DNA. GeneRuler (Fermentas) was used as DNA ladder mix.

**Fig. S5.** Binding kinetics of CRP-$His_6$ to *iol* promoters analysed by SPR spectroscopy. Biotinylated double-stranded DNA fragments comprising the promoter regions of $P_{iolR}$ (A),

$P_{reiD}$ (B), $P_{iolT1}$ (C), $P_{iolT2}$ (D), $P_{iolG2}$ (E), $P_{iolD1}$ (F), $P_{iolI2}$ (G), $P_{iolA}$ (H), $P_{iolC1}$ (I), $P_{iolC1.2}$ (K), $P_{iolE}$ (L), and $P_{argS}$ (M) were captured onto a streptavidin-coated sensor chip, and different concentrations of purified CRP + 20 µM cAMP [2.5 nM (purple line), 5 nM (dark blue line), 7.5 nM (green and violet line), 10 nM (blue line), 25 nM (yellow line), 50 nM (orange line), and 100 nM (red line)] were injected over the chip. Association rates (*ka*), dissociation rates ($k_d$), the overall affinities ($K_D$) and the binding sociometry were calculated and are depicted below each sensorgram. All sensorgrams are representatives derived from three independently performed experiments. n.d., not detectable.

**Fig. S6.** Transcriptional activities of *iol* gene promoters in the absence of *ssrB*. Luciferase reporter fusions to the promoters of *sseA*, *iolE*, *srfJ*, and *iolR* in plasmid pDEW-201 were tested in 14028 and in 14028 Δ*sseB* in LB and in AMM. The transcriptional activities were measured as RLUs at an $OD_{600}$ = 0.7. All experiments were performed independently thrice; standard deviations are indicated. Significant differences ($p < 0.01$) are marked by asterisks.

**Fig. S7.** Growth curves of *S*. Typhimurium strain 14028 and its mutants 14028 Δ*ssrB* and 14028 Δ*ssrAB* in MM with MI as sole carbon and energy source. The long lag-phase of approximately two days as indicated by an arrow is a characteristic feature of strain 14028 growing under these conditions. Monitoring of the $OD_{600}$ was done for 76 hours by an Epoch reader; standard deviations of three independently performed experiments are shown.

**Table S1.** CRP binding consensus sequences.

**Table S2.** Oligonucleotides used in this study.
