# Supplementary figures and images for: Hierarchic regulation of a metabolic pathway: H-NS, CRP, and SsrB control myo-inositol utilization by Salmonella enterica
Source: Microbiol Spectr. 2023 Dec 14;12(1):e02724-23. doi: 10.1128/spectrum.02724-23 (PMC10783015; doi:10.1128/spectrum.02724-23)

FIG. S1

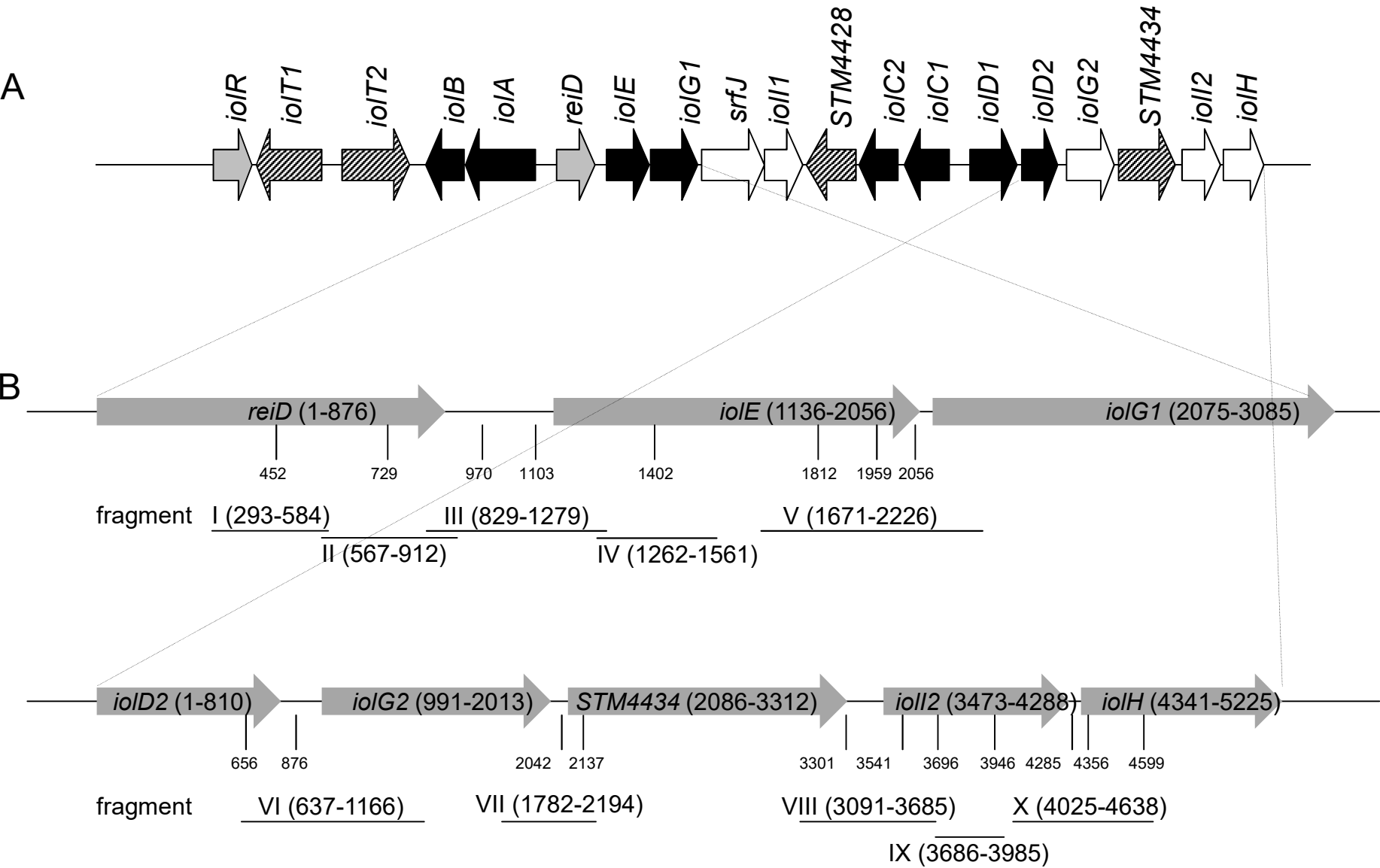

FIG. S2

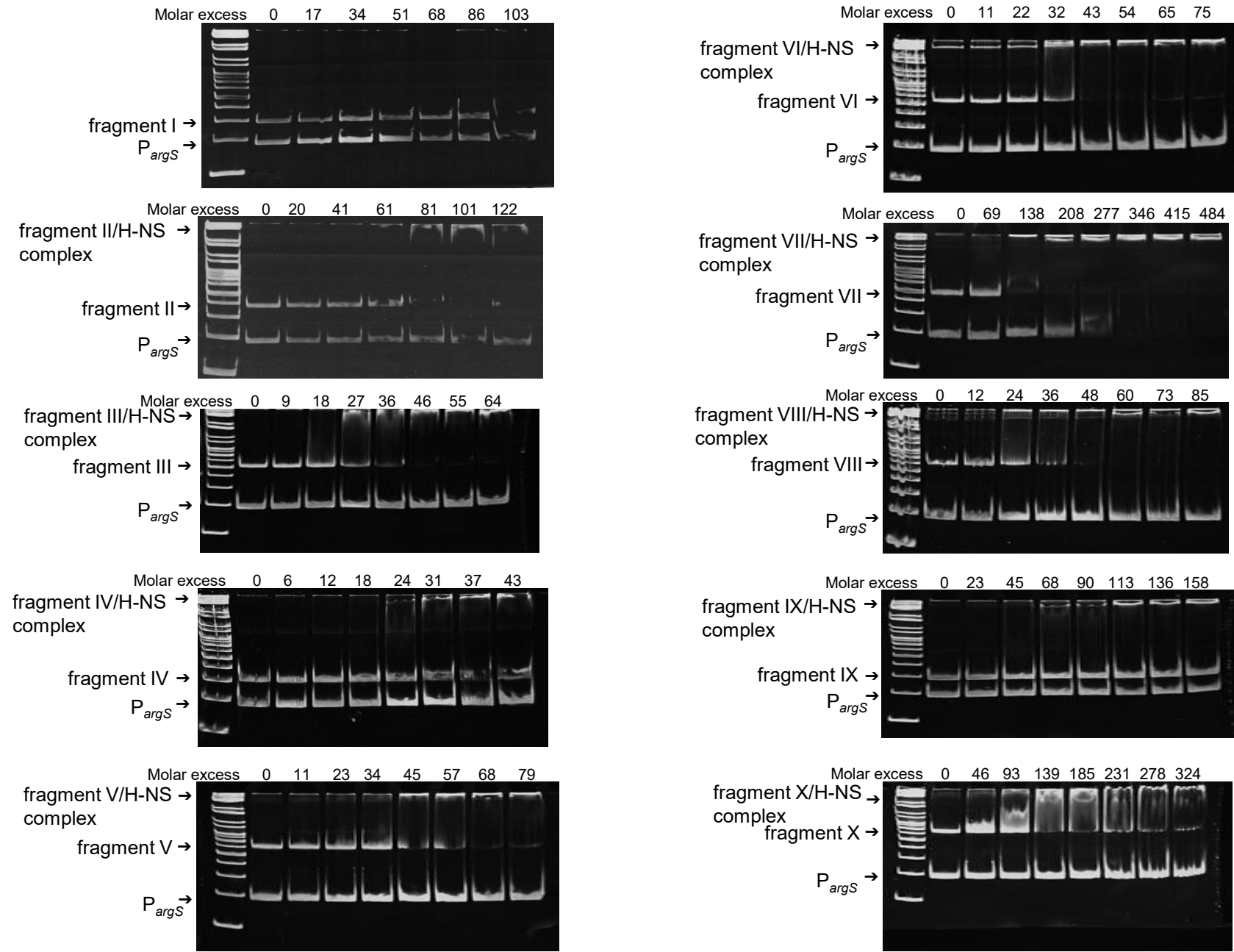

FIG. S3

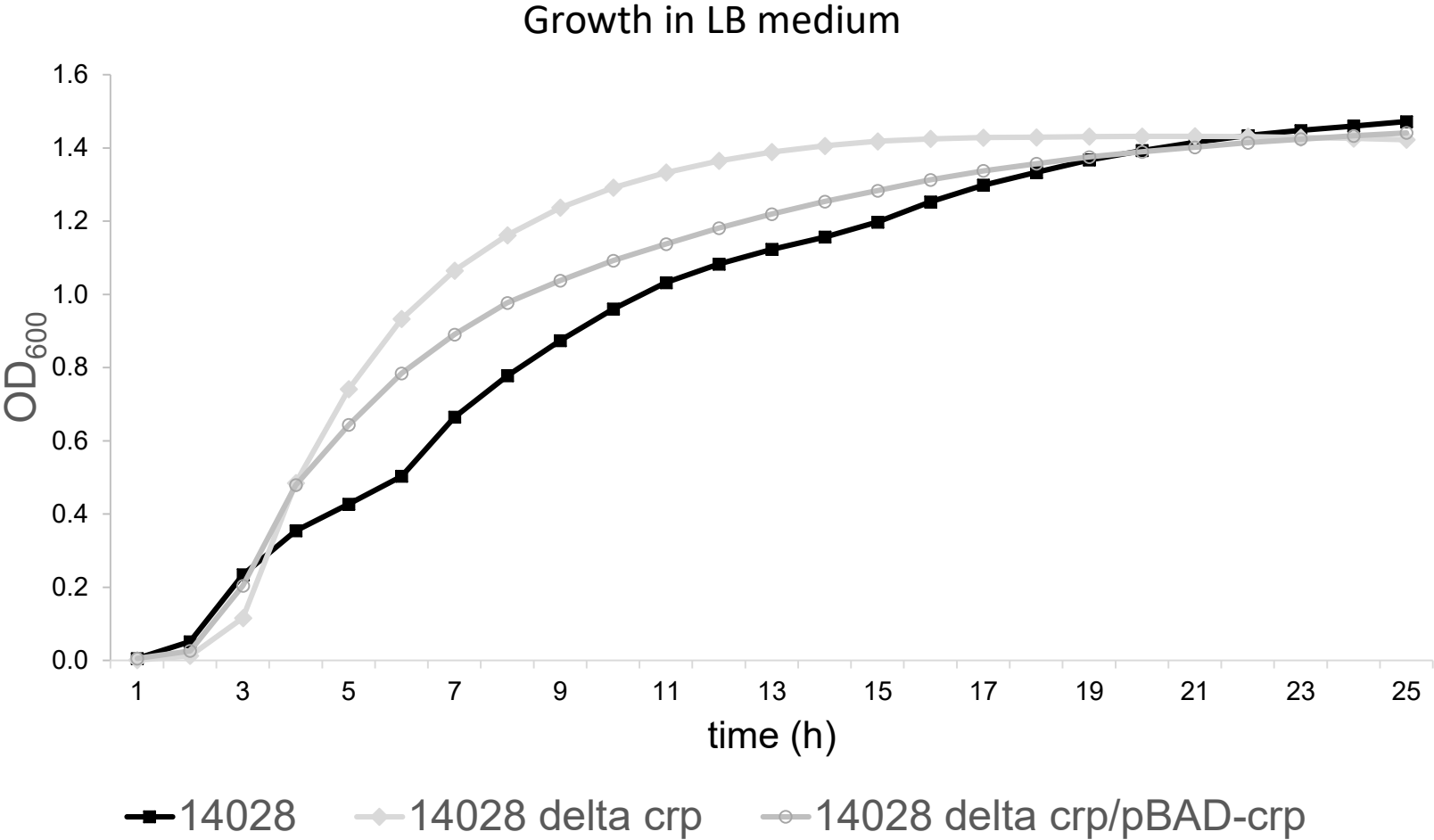

FIG. S4

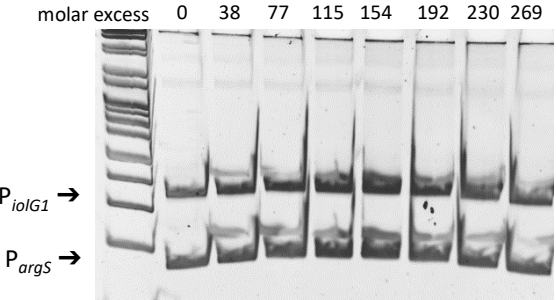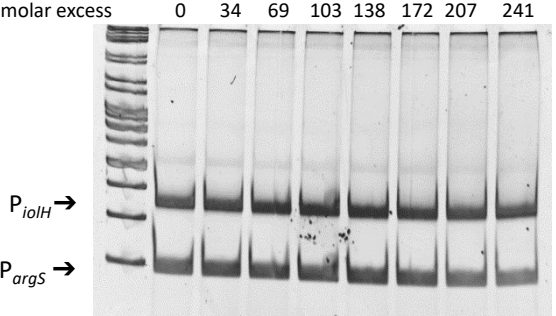

FIG. S5

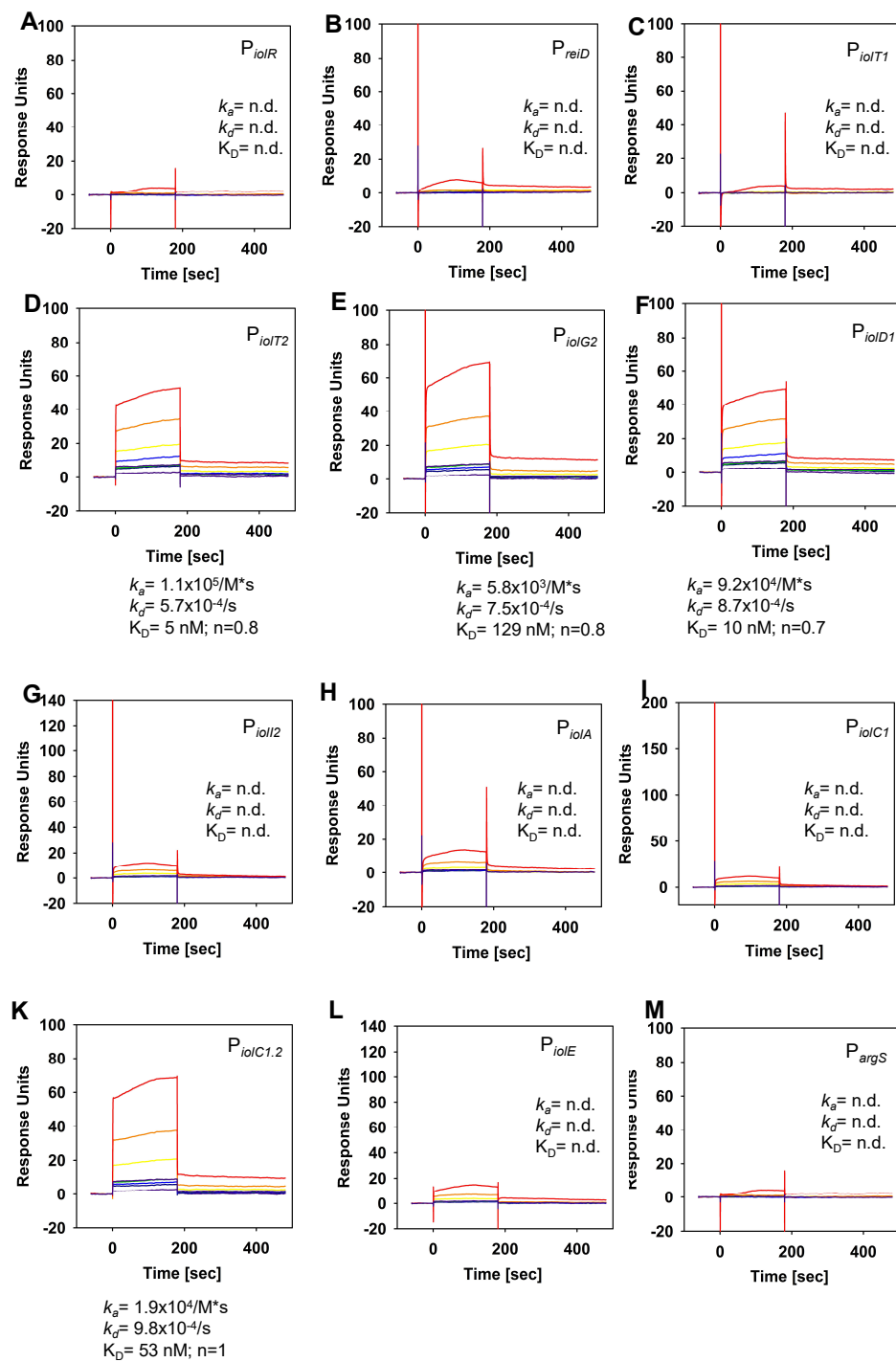

FIG. S6

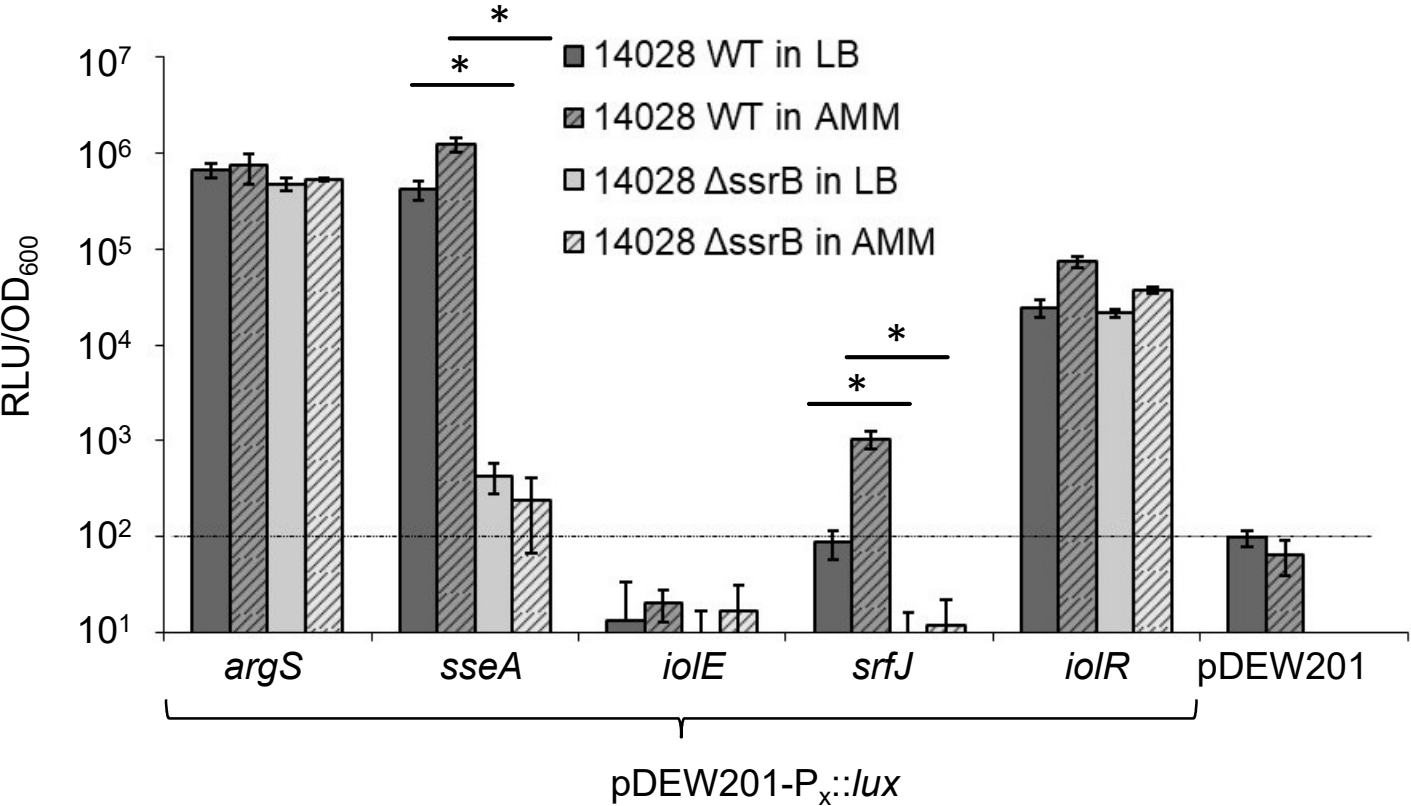

FIG. S7

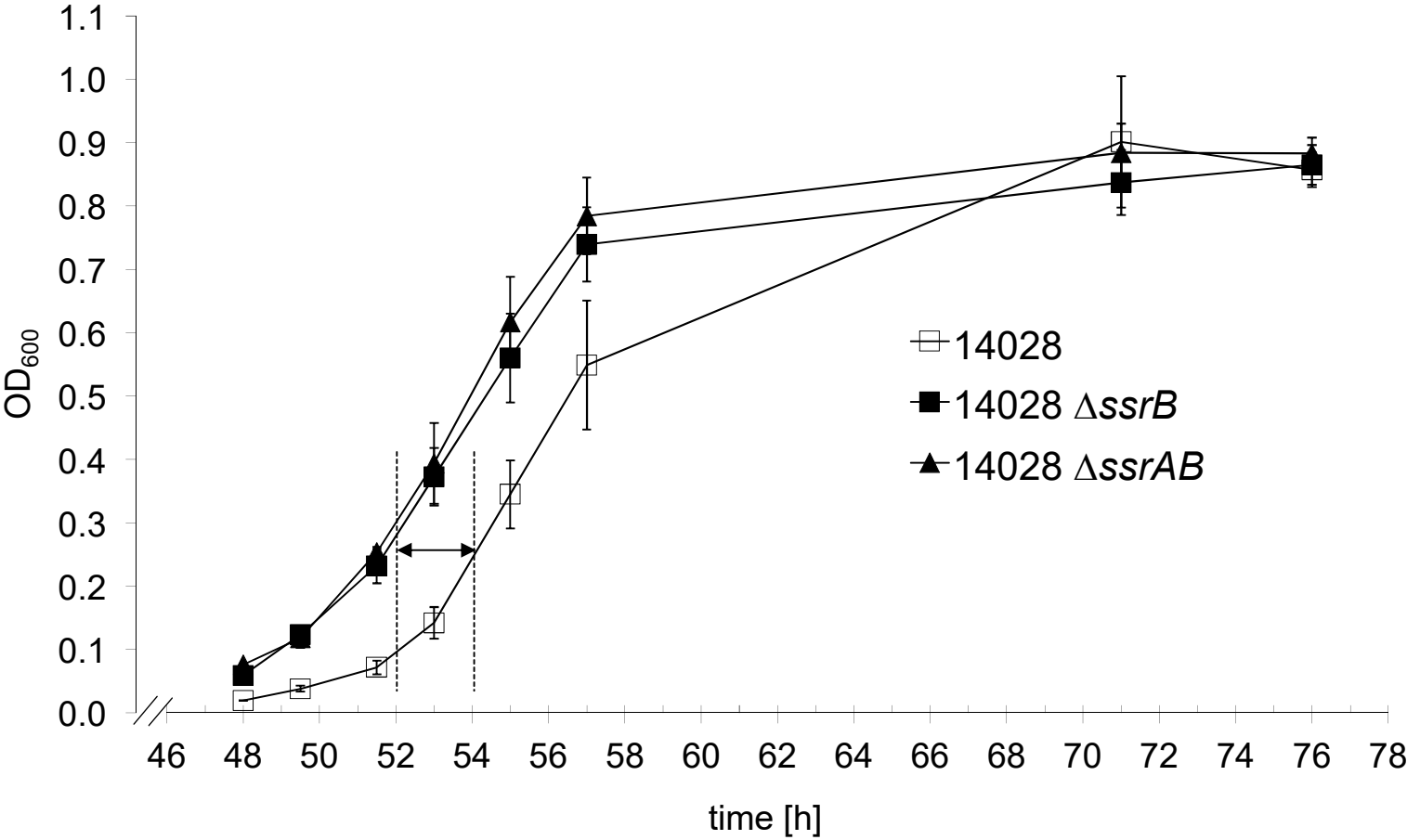

Supplement: Fig. S1 to S7 — Supplementary figures. [file spectrum.02724-23-s0002.pdf]
