# Supplementary material for: Hierarchic regulation of a metabolic pathway: H-NS, CRP, and SsrB control myo-inositol utilization by Salmonella enterica
Source: Microbiol Spectr. 2023 Dec 14;12(1):e02724-23. doi: 10.1128/spectrum.02724-23 (PMC10783015; doi:10.1128/spectrum.02724-23)
Supplement: Table S1 — MI-island CRP sites. [file spectrum.02724-23-s0003.doc]

Table S1: CRP binding consensus sequences.

Xxx: CRP binding consensus sequences; xxx: start codon; xxx: stop codon

>mi-island

**STM4416**

ATGCGCATTCATATTTTGGGAATTTGTGGCACTTTCATGGGGGGGCTGGCGATGCTGGCTCGCTCGCTTGGTCATGAAGTAACGGGTTCGGACGCCAATGTGTATCCGCCGATGAGTACCTTACTTGAGAAGCAAGGCATTGATCTGATCCAGGGTTATGACGCCAGCCAGCTCGATCCGCAGCCGGATCTGGTGATTATCGGCAATGCGATGACGCGCGGGAATCCGTGCGTGGAAGCGGTGCTGGAAAAAAACATTCCCTTTATGTCTGGTCCACAGTGGCTGCACGACTTTGTGTTGCGCGACCGCTGGGTACTGGCGGTCGCCGGTACCCACGGCAAAACCACGACCGCAGGCATGGCGACCTGGATACTGGAAGCGTGCGGGTACAAACCGGGCTTTGTGATTGGCGGCGTACCGGGCAACTTTGAGGTTTCCGCGCGCCTGGGAGAGAGCCCGTTCTTTGTTATCGAAGCGGATGAGTACGACTGCGCGTTCTTTGATAAACGTTCTAAATTTGTGCATTACTGCCCGCGGACGCTGATCCTCAACAACCTTGAGTTTGATCATGCCGACATCTTCGACGATCTGAAAGCGATCCAGAAGCAGTTCCACCATCTGGTGCGCATCGTGCCGGGGCAGGGGCGCATCATCTGGCCGGAAAACGACATCAACCTGAAACAGACCATGGCGCTGGGCTGCTGGAGCGAGCAGGAGCTGGTGGGCGAGCAGGGACACTGGCAGGCGAAGAAGCTGACTACGGATGCTTCCGAGTGGGAAGTGTGGCTGGACGGCGAAAAAGTCGGCGATGTGAAATGGGGCCTGGTCGGCGAACATAACATGCACAACGGTCTGATGGCGATTGCCGCCGCGCGCCACGTGGGCGTCGCGCCCGCAGAAGCGGCCAGCGCGCTGGGGTCATTTATTAACGCCCGCCGCCGTCTGGAGCTACGCGGCGAAGCGAACGGCGTGACGGTGTATGACGATTTTGCTCACCACCCGACGGCAATTCTGGCCACGCTGGCGGCGCTGCGCGGTAAAGTGGGCGGCACCGCGCGCATTATCGCAGTGCTGGAGCCGCGTTCTAACACGATGAAGATGGGGTTATGCAAGGATGACCTGGCGCCGTCGTTAGGGCGTGCGGATGAAGTGTTCCTGCTGCAGCCGCCGCATATTCCGTGGCAAGTGGCGGAAGTCGCTGAAGCCTGCGTACAGCCTGCTCACTGGAGTGGCGATGTCGATACGCTGGCGGAAATGGTGGTGAAAACCGCACAGCCTGGCGATCACATCCTGGTGATGAGCAACGGCGGTTTCGGCGGCATCCATCAGAAATTGCTGGATGGGCTGGCGAAAAAAGCTCAGAACGTAACAGCGTATTAAATGACTATGCGGTCAGCCAGCCTTGTGGCTGGCTGTTTTATATGGGGCATCTTGCCATCGAAAGCGCATTCCGCTCGCCGGGAGACGTTAAAGCAATACTGAAAAATCATTTGCCGCTCAGTATTGAAAAAGAGTAGACGCATATCCTTAAAGTAATCAGTGTAATACACTAAGAGTGTTCCGCCGGTTGCCGTATGCGGGACGAAAAGAGCCAGTTCGCAATAACAGATGAAGGTTTCGTGATTTATAAACGTCATCTTCTGCCTTTCAACGTTTGCGATGCCGCCTGGCTGCGGGCATCGTCCAGTCATAACAATGCTGATCCTGTCGCATTTATGCGGTCAGATTCAGATTGCTCAGAACCCAGCCCGCCAGCAAATTCTGTACTGAAGGTAACCACAGCGCAATTTGAATGTTGTTAACTGTATGTTCAGTTCATTTGTGCTAATATGGTTATTTACGAAATTTTCGTTCTATTAGAGTATCATGC

***iolR*** ATGTCTAAACATCAAACTCAACTTTCCTTACTGCAGGATGATATCCGCAGTCGCTATGACAGCCTTAGCAAACGTCTTAAACAAGTTGCTCAGTATATTCTGGATAACAGTAATAGCGTTGCTTTTGACACGGTAGCTTCCATCGCGCAACATGCCGATGTTCCCCCTTCGACACTGATTCGTTTTGCTAATGCGTTTGGTTTTAGCGGATTTAATGAAATGAAACAGGTATTTCGCCAGCACCTGATGGAGGAAACAGTAAGCTATACCGAGCGGGCGCGTCTGTTCAGACAGAAAAATGCGGATGAAGGGGAACCTACCCCGGAAAAGCCGGGTGAAATTCTGAAGCTATTTTCAATGGTGAATGCACAGGTATTACAACAATTACCGGTGCAGATTAAAAATGAGCAGTTGGATGCGGCGGTAAATCTGTTAGCGAAAGCCGACAATATTTATGTGATTGGATTACGTCGTTCGTTTAGCGTGGCCAGTTATTTAACCTACGCATTGCGTCATCTGGAGAGACGGGCATTTTTAATTGACGGTATTGGCGGAATGTTCTCTGAACAGCTAAGTATGGTCAGCCCCGACGATGTCGTTATTGCCATCAGCTACTCGCCTTACGCGCAGGAAGTGGTGGAATTAGTCGAGCTTGGCGCGAAACGCGGTGCGCACCATATTGCGATAACCGATAGCCAGGTCAGCCCGCTGGCAGCGTTCAGTGAAGTCTGTTTTGTGGTACGTGAAGCGCAGGTCGATGGTTTCCGTTCTCAGGTCGCGTCCATGTGTCTGGCGCAAACGTTAGCGGTTTCACTGGCGCTGGCGACGGAGTAA

AAAAACAAGCCGATGATCGTATTTAGCGATCATCGGCATTGTGGTGAAAC

TTAATCAGAATAACGTTCGGTTTGAATTGGCTGCAGTTTTTTTCTGCGTTTTGCTAACACTACGCTCTCCATTTTCTCCAGCGAAATGCCTTTTGTTTCCGGCAGGTAACGGCAGATAAAGAAGTAACTGAAGATGCAGCAGATGGCAAATATCCACATTGGAAATGCGCCGTGGAAATGCGAAAGTAAATACGGGTTTTCATTAATCATCGGGAAGAATTGCGAAACTAAAAAGTTTGCCATCCACATAAATCCGACCGAAATACTCATTCCCTGCGACCTCATCCGGTTAGGGAATATTTCAGAAATAAGCACCCATGCGCCCACGCCCCAGGAAAGCGCGTAGAAAATCATAAAGAACAGCATACCGAATAGCGCGAAATAACCGGTTGCCTGGCTGTACAGCGCCCATGAGGTGAGCAACAAGCCGATGATGCTGCCGATAGTGCCTTTACGCATCAGCGACAAACGCCCCATCTTATCCATAATCATTGCGCCAATAATGGAACCGATAAGCTGAATCACGCCGATCCAGATTGTCTGGAACAGGGCCTCCTGGGCGCTACCGGTCACATCCTTAAGGACGATTGGCGCGTAATACATCATGACGTTAACCCCTGTTACCTGTTGCAGCATAGCGATCATGCAGCCCAGGATCAGGATGAAACGCACGTTCCCGTCGCGGTAATTGAGTTTTTGATGGGCGTTTAGTTGGTCATTTTGCAGTGAGGTTTTTATATCGGCAAGAAGATGTCTGGCATGTTCTTCGTTGGATATTTTGGTCAGTATTTTTAGCGTCTCCTCCTCGCGACCAATCATCATCATCCAGCGGGGAGATTCCGGGATTAAAAAGACTAAAATACAGAACAAAATGCAGGGAATAATACCCGCAGCAAACATATAGCGCCAGCCAAGTTCAATGAGCCAGGTATCCGCTGCAATCGATGCTATTTTGTAGTTAACGTAGAAGATCAGAATCTGACCGAAAACGATAGCGAACTGCTGCATACTTAATGCTCTGCCGCGCATGTTTTTCGGTGAGACTTCAGACATATACATCGGTGATACTGTTGCCGCCAGGCCGACTGCCAGTCCACCGATAATGCGATAAATCACAAAATGGGTGAACGTGTAGGAGAGGGAGGTGCCCACTGCGGAAATAGTGAATAACAGCGCTGAAACCATCAATGATTTCTTGCGTCCAAAACGTTTGGATAAATATCCGGCGCTAAACGAACCAATAACGCACCCCACCACAACGCAGGAAACCGCCCAGCCGGTCTCAGCAGGAGATAGATGAAAGTAGCTGGTCAGTGAACCAATCGCACCTGAAATAACAGCAGTATCGTAGCCAAATAATATTCCGCCCAGTGCGGCAATCGCGCAGATGCGTAATATGTAACCCGTATTATAACAACTATCTGATGTGGACAT ***iolT1***

AGGTAGATTATCTCCAGAATAAACTTCATCTATCTGTTTGAATTTAGTGAACAAATAGACAAATTCATCTTATGTCGGTCATTGCCGTAGCGCTGCCGACGATCTTATAGCTATTGAGAACTCTCGTTTCACAACCTATGTTTTAATTTCAAAACGATCAATAATGAAACTTATGTTTTGTTATGGGTATCACATTTCGAATTTCATAATCCTGGCGTTTTTTATCGTTAAGATGCTGCGTTTTACGCAGTGCTCTCCTCTATCTTGATGAAGTTACTTGATTTTATTGATTTCGCGACAGTACCTGAACTCAATTTGTCAGGGGCCGTACTTTTTGTTCTTTCCTGGAACATCTCCATTTCGTGATCTTTTGCATGGAATTTTTCTTCTAATGAATGCAAAAATAAAATGAAAAATTTCATTGGTGTGTTGTTTCCTCAACAGGCAAGGGAGGGATT

***iolT2***

ATGTCTCAGAGAAGTAAGTACAATTCGGCCTATGTGTACGTCCTGTGTTGTATTGCGGCGCTGGCTGGATTGATGTTTGGTTATTCAACGGCGGTGATTACCGGAGTGGTATTGCCTTTACAGCAGTATTACCAACTGACGCCAACCGAGACCGGATGGGCCGTTTCCAGTATCGTGATTGGTTGTATCATCGGCGCGCTGGTCGGTGGAAAAATTGCCGATAAACTGGGGCGTAAACCTGCGCTTCTGATCATTGCGATCATTTTTATCGCTTCTTCCTTAGGGGCGGCGATGAGTGAATCGTTCATGATCTTCTCCCTTTCCCGCATTGTGTGTGGTTTTGCGGTTGGGATGGCCGGAACGGCATCCACCATGTATATGTCTGAACTGGCGCCTGCTGAAATTCGCGGCAAAGCGCTGGGCATTTACAATATCTCCGTGGTATCTGGCCAGGTTATCGTGTTTATAGTCAACTATCTGATAGCAAAAGGAATGCCTGCTGATGTGCTGGTTTCCCAGGGCTGGAAGACTATGCTTTTTGCCCAAGTGGTACCCTCCATTGCGATGTTAGCGATTACGCTTTTCCTACCCGAATCACCGGCATGGTGCGCCCGTAACAACCGCAGCGAAGCTCGTTCGATAAAGGTGCTTACCCGGATCTACAGTGGATTAACGGCCACAGATGTGGCCGCTATTTTTGACAGCATGAAAGAAACCGTACGTTCACAGGACAACGTCGCCGGGGGAGAACGCACCAACCTGAAAAGCTCGCCGGTGCTCCGCTATATTCTGTTGGTTGGATGCTGTATCGCCGTTTTGCAACAGTTCACAGGCGTTAACGTAATGAACTATTATGCGCCGCTGGTGTTGCAGAACAGCAGTACCGAAGTGGTTATGTTCCAGACCATTTTTATCGCGGTATGTAATGTGGTGGGCAGTTTTATCGGCATGATCCTGTTCGACCGCTATGGCCGTATACCGATTATGAAAATTGGTACCATCGGCTCAATTGTCGGCCTGTTGATCGCGTCATACGGTTTGTACACCCACGATACAGGCTACATTACCATCTTTGGCATCCTGTTTTTTATGCTGCTGTTTGCCGTCAGCTGGAGCGTTGGCGCATGGGTACTGATTTCTGAGGTTTTCCCTGAAAAGATAAAAGGTTTTGGGATGGGGCTGGCGGTGAGTCTGATGTGGATAGCCAACTTCCTCATCTCACTGTTGTTCCCGGTCATAAATGATAACGCCTGGCTGCAGGAGACCTTCGGCGGCGCTTTCTCGATGTGGATTTTTGTCGTCTTTAATTTGGTCTGCTATGTCTTTATTTCTCGTTATGTGCCGGAAACAAAAGGGGTGCCGCTAACAGAAATTGAACGGCTGGCCGAGAACAAGCTGCGTGAAATTCAGGGGAAACGTCGCGATGTAATAGCCTGA

TACCTCTCCAATATTAGCGGAGAATATCCAGAGATATTCTCCGCTGGTTACACAACCTTTACTCCCTCCAGCGAGTGCGCACAGGATATACCCTGTTGAAACCTCCCCAGTGTTTACCACCTCTACGTAACTAATTGATCCATAATAATATCGGCAAATAGGGTAAGTCTTCTTCTGCATCATTATTTTATTGCGTATTCCTGGTGGGGGCCGTGCCGTAAATACGCTATATACAAAACAGGGAAATAATTTAAGAGAAGGCGGATAGCGATCTTATGAGCGTGTGTGGCGCAGAGTTGAATAAAAAAGCCAGCCGCAGAGGACGGCTGGCAAAGTACCACTCAACGAAGTGTTATCGCGGGTAGTCTGGTGAGTTAATCCACGCGTGGTTCTCTTCCCAGGTGAAACGCCATTTTCGTAGCGGGCCGGCCATCACGTTCAGATAGTAGTTATCGTAACCCGCGATGGTGGCAACCGGGTGGTAGCCCTTAGGTACTTTCACTACATCCCGGTTATAGACCGCCATACATTCATCCAGGCTGCGATCGTCGGTGTAGACGCGCTGTAAGCAGAATCCCTGAGGTGGATTGAAACGGTGATAGTACGTCTCTTCCAGATAGGTCTCCTGGCCTTCAACTGCCGTATCGTGCTTGTGCGCGGGCCAGGAGCTGGTATCGCCTTCATTAGTGTACACTTCAACGACTAGCAGGCTGTCGGCGAGCTGGCTATCAGGAAGGATGTTATGCACCAGGCGCTGATTGCGCCCTTTTCCCCGATGCTCTACCCCGACCTCTTGTGGCGAGATCAGACGTACCGGAAGCTCTCCGAATCCCGGCGCGCTGCATACGGCCAGCTCAAGGTCGGTTTCGGCTGTCACTTTCGCCTCCGTATGATGGGGGAGGTAAACCGAATAAGCCGGAATGCGTTCAAACGGACTCATGCGCTGACCAATCCGATAGAAAAAACTGTCGGCGGCCTTAACGCTGGCCAGCCCGGCAACCAGCACCAGACAGCGTTCACGCTCATCGGACGGCAGGGTTATCGACTCTCCAGCCTTCAACTGCCAGACATCAAAACCGACATACTCCCAGCCTGCATTTTGAGGGGAAATGTGTTGGATATTTCCCGACTCAGAGGTACAGGTACTCAGCAAGTTGGCCAT***iolB***

AATCTTCTCCTTCCTGAAAGCGTA

TTAACCGAGAGTCGGCATACTGAACTCCGATACGATCTGCTGTCCGTTCGGCCAACGCGAGGTAACGGTTTTCATCCGGGTATAGAAGCGCACGCCATCCGGCCCATGGACGTTCAGGGCGCCAAACACCGAGCGCTTCCAGCCGCCAAAAGAGTGAAATGCCATCGGAACCGGAACTGGAATATTAATACCGACCATCCCGGCTTCAACCGACTGGGCGAATTCGCGCGCATAGTGACCGTTGGTGGTGAAAATCACGCTGCCGTTGCCGAATTCATGGGAGTTTACCGTGCTAATGGCGGTGGCATAATCCGGGACGCGCACAATTCCGAGAACCGGGCCAAAAATTTCTTCACGCCAGATGGTCATCTCAGGCGTGACGTTATCAAACAGAGTGCCGCCGACATAAAAGCCTTCAGGATATCCCGCCACGCTATAGTTGCGGCCATCCATGACCAGAGTTGCTCCCTCGCTCACCCCTTTGTCGATGTAACCCAACACTTTCTGTTGATGAGCACGTGAAACAACCGGGCCCATTTCATTTTCATCTTTGCCGCGCAGCTCTCCCGGGCCGATACGTAGCGCCGCAATCAGCGGTTTCAGGCGTTCGATCAGACGGTCCGCCGTTTCATCGCCCACCGCGACCACTACCGGCAGCGCCATACAGCGTTCGCCCGCGGAACCAAATGCTCCGCCCATAATGGCGTTGACGGTGGCGTCCAGATCTGCATCCGGCATGACGATTGCGTGATTTTTGGCGGCCCCGAAGGCCTGTACGCGTTTGCCATGGGCGCTGGCGGTGGTATAGATATGTTCCGCGACGGCGGAGGAACCGACAAAGCTGACGGCCTGTACGCGAGGGTCGCTGGTCAACAGGCTAGCCGCCTCATTGCCACAGTGAACAACGTTAAACACGCCGTCCGGCAGGCTAGCCTCCTGCAGAAGCTGCGCCATACGTAACGATGCGGACGGCACCAGTGCGGGCGGTTTCAGGACGAAGGTATTGCCGCAAGCCAGAGCAACCGGGAACATCCACATAGGCACCATTGCCGGGAAGTTAAACGGTGTGATGCCTGCCACAACCCCAAGCGGCTGCATTAATGAAAAGCTGTCAACGCCTGAGCCGACGTTAAAGGAGTACTCTCCTTTGCTGAGGTGCGGGATCCCGCAGGCAAATTCGACAACCTCTATGCCGCGCGTCAGCTCGCCAAGCGCATCTGACCAGACCTTGCCATGCTCGCTGACGATCAGCGCCGCCAGTTCGTCCCGATGCTTTTCCAGTAATACTTTGAATTCAAAAAGGATACGTGCCCGGCGTAGCGGAGTCATTTTACTCCATGCCGGAAAAGCCTCATGTGCGCTTTGAATTGCCGCCAGCATCTCCTCTCGGGTGCTCTGGGTGACCTGACGAATTACTTTACCGGTGGCCGGATTAGTCACCGGTAGCGTCTCGCCGCTGCTGCTCAGTGTCGTTTTACCATGAATAAAATTGCCGACTGTTTCCAT ***iolA***

CTCTTTTTTATCCTCTTAAACCGATGTTGACGACGTTCTGACTGAGCGGTCACAGGAAAGCCTGGTTATCTTCAGCCTAAAACGCATACCGTATTCAATGTAAATTTTAGGATTAAAAATTTCAAATTTATTTTTATGAAATTTTATTTTGAAAGTTTGACGGCTGATTTTTATACGAGGGTATGTGCAGCAAGAAATCAAAAATATAATTAACTTATTGATTTTTATCGTTTTTATTTGGTGTGTCATCAATGGTTGTCTGTTGTGACGAGGAAAGATTTGGAAAAAATTATGATCTATATAGCATTTTTGTTTCATTTGGTGTGGTATAAAAATTCAAATTTCAGGGGGGTTTCGGCACCACAGTTTCGGATATTGGAGGGTAAATCATGCGCTTGTGCTTCTGTCCTTCAGAACGAGAGAATAAGGCTCAGCCCCAGTTGGCGTATGTGAGTCAGGTCAGTGAGAGCAATGGTCCGTTTGTCCGGTTCATGCATATGCACCCACAGACCGTTGAACTGATTCTTATCACCGAAGGAGACGGGGAGTATTTCATTGGCGATCGCATCTACCCAGTTCGTAAAGGCGACCTGGTCATTTATAACAGCCAGGTGGTGCATGATGAGTATCTGGAAAGCGGGCGGCCAATTGGCACTATTTGTTGTGGGATTAACAATATTTCGTGTCCGGGTCTACGTGAGAATGCGCTAATACCGGATGATATTGTGCCGGTTATTCCACTGTATCACCATTATGCGACGGTGGAAAAACTGATGAGCTCCGTGTTTACCGTTATTAATCAGCATGCGGTAGAAGGGCCAGCAATGGCGCAGCTATTGACCCAGGTAGTACTCAAATACATTGAGGGAAATGTCTTATTGCGCACAGGTAACGATGCCATACAGCGCCATGAAAATTTACTTGATTCAATAAAATCCTATATTGATCGCAATTTCTATGAGCCAATTCGGCTGGATACGCTGGCGACGAAATTTAATGTCAGTCCTTATTATGTTTCCCATGAATTTAAACGCCGCTATGGTTATTCACCGATGGATTACCTGATAAAACGTAGGCTGGGGGAGGCGCAATCGTTACTAACCACGGATGAAGGCGGGCGCGAAAAGATCACCTCTATTGCTTATCGTGTTGGCTTTAGCAACCTTAGTCATTTTCAGAATTACTTCAAAAATAAAGTAGGGAAAACGCCCGGTCAATATCGCAAGGACTATCGTAAAGCTAATTATTTATTATTTGAATATTAATGCTTGCTAAAATTTAATCGTCATGAGAATGGTCGCAATACTGCGGCCATTTTTTATTTGTCGGTATATTTTATGCGTTTATCTATAGCGCGAAAAGTCTGGCAATATCATTAAAGAGTGATGATTTTTTTGTGATACCTGACGCATTTATCCCACTTCTTCAACCAGGATAGATAAAGTTTTTCAGGCTACAAAAAGTAATATGTTGTCCATCAAAGGTTAATTTGCATCGCGGTATGCAGTAAAAGCGTTTCATTAA

***iolE*** GTGGGAGCCAGCAATATGTACAATGTAAAAAAGAGCATTAAGTTGGGGATTGCGCCTATCGGTTGGCGCAATGATGATATTCCTGAGATTGGTAAAGAAAATACATATAAACAAATACTTAGTGATGCCGCGCTCACCGGTTTTTCCGGGACGGAAGTTGGCGGTTGTTATCCGCAGGATCCTGCTGAGTTAAATAAAGAGTTAATGCTTCGCGGACTGGAAATACCAGGCCAGTGGTTTTCTTCCTTTATTATTCGCGATGGTATTGCATCTGCGATGAATGCTTTTGAACAGCATTGTGCTTATTTGCAGGCAATACATGCATATGTCGCCGTTGTTTCTGAACAAACTTATAGTATTCAAGGCATTATCGATAAGTGCGTTTATACAGAGAAGCCTAACTTCAGCGACAGCGAATGGCAGCTTTTATGCGAAGGACTTAATGCGCTTGGGAAGATTGCCAATGCGCACGGGCTGAAGCTCGCTTTCCATCATCATATGGGCACCGGTGTGCAGACTCTGCCAGAAGTGGATCGACTCATGGAGAATACCGATCCGCAATTTGTACATCTGCTGTTTGATACCGGGCATATATATGTTTCTGACGGCGATGTCATGCCGCTACTGAGTAAACATTTTGATCGTATTAAACACGTTCATTTTAAAGATGTGCGTAATGAAAAACTCAAAGCATGTCGCCTCGCGAAGAAATCATTCCTCAATTCTTTCCTTGATGGTGTGTTTACCGTCCCCGGCGATGGAAATATTGATTTTAAATCCGTATTAGCTTATCTGGTCGGGCATCAATATTCTGGCTGGATTGTTGTTGAGGCCGAGCAGGATCCTAAGAAATATAACCCATTGGAATATGCGCAAAAAGGTAAAAAGCATATTGATGAGTTACTGAAAAATTATCTTTAA

TTGAAAAGGAAACAGAAA

***iolG1***

ATGACTTTAAAAGCAGGTATTGTAGGTATCGGCATGATCGGCTCCGATCACTTAAGACGTCTGGCCAACACCGTGTCGGGTGTAGAGGTTGTTGCCGTATGTGATATCGTCGCGGGTAGGGCGCAGGCGGCGCTGGATAAGTATGCGATCGAAGCCAAAGATTATAATGACTATCACGATCTGATTAATGATAAAGACGTTGAAGTGGTCATCATTACCGCATCAAATGAGGCGCACGCCGATGTAGCCGTTGCCGCGCTAAATGCTAACAAATATGTTTTCTGTGAAAAACCGCTAGCGGTGACGGCTGCGGATTGTCAGCGTGTGATTGAAGCAGAGCAGAAAAATGGTAAGCGCATGGTGCAGATTGGTTTTATGCGCCGCTACGACAAAGGTTACGTTCAGCTGAAAAATATTATCGACAGCGGCGAAATCGGTCAGCCATTAATGGTTCATGGACGTCATTACAATGCCAGCACGGTGCCAGAATACAAAACGCCACAGGCTATCTATGAAACATTGATTCATGAAATTGACGTTATGCACTGGCTGCTTAACGAAGATTATAAAACCGTTAAGGTTTACTTCCCGCGCCAGTCCAGCCTGGTGACCACTCTGCGTGATCCGCAGCTGGTTGTAATGGAAACCACCTCCGGCATCAATATTGTGGTTGAAGTATTTGTGAACTGCCAGTATGGCTACGACATTCACTGCGATGTTACCGGCGAGAAAGGAATGGCGGAGCTGCCAACCGTCGCCAGCGCCGCGGTACGGAAAGCGGCGAAGTATAGCACCGACATCCTGGTTGACTGGAAACAGCGCTTTATTGATGCCTACGACATTGAGTTCCAGGATTTCTTCGATCGTCTGAACGCAGGATTGCCGCCGGCAGGCCCAACGTCATGGGATGGTTATCTGGCTGCGGTTACCGCTGATGCCTGCGTGAAGTCTCAGGAAACAGGGAATACCGAGATTGTTGAATTACCCTCAAAACCTGATTTCTACAAATAA

ACCTTCCGCGTATTAAAACTCCTGCCGGATAGCGATGCTTAAGCGTCCTATCCGGCATATCACAATTTATTATCTTCTTTTATCCGGAACTTCCCT

***srfJ***

ATGAAAGGCAGACTCATCTCTTCCGATCCGTATCGTCAGCAATTCCTTGTTGAGCGTGCGGTCTCTTTTTCGCATCGTCAGCGTGATTGCAGTGAATTAATCAGCGTCTTACCGCGCCACGCGTTACAGCAGATTGACGGATTCGGCGGCAGCTTTACCGAAGGTGCGGGCGTGGTATTCAACAGCATGAGCGAAAAGACGAAGGCGCAATTTCTTTCCCTTTATTTTTCTGCTCAGGAACATAATTACACTCTGGCGCGGATGCCAATTCAGAGCTGTGATTTTTCCCTGGGCAATTACGCGTATGTCGATTCCAGCGCTGACCTGCAGCAGGGACGGCTCTCCTTTTCCCGCGATGAAGCGCATTTAATACCGCTGATTTCCGGGGCGTTGCGGTTAAATCCACACATGAAGCTGATGGCTTCGCCGTGGAGTCCGCCGGCGTTTATGAAAACTAATAACGATATGAACGGTGGCGGCAAGCTGCGGCGCGAATGCTACGCCGACTGGGCCGATATCATTATCAACTACCTGCTGGAATACCGCCGCCACGGCATTAATGTGCAGGCGCTCTCCGTGCAGAATGAGCCGGTGGCGGTAAAAACCTGGGACTCCTGTCTGTATAGCGTGGAAGAGGAGACAGCCTTTGCCGTGCAGTATCTGCGTCCGCGCCTCGCCCGGCAGGGTATGGATGAGATGGAGATCTATATCTGGGATCACGATAAAGATGGCCTGGTGGACTGGGCTGAACTCGCCTTTGCTGACGAAGCTAATTATAAGGGAATTAACGGGCTGGCATTCCACTGGTATACCGGCGACCATTTTTCGCAAATACAGTATCTGGCCCAGTGCCTGCCGGATAAAAAACTCCTGTTTTCCGAAGGCTGTGTGCCAATGGAGAGCGATGCCGGTAGCCAGATTCGCCACTGGCATACCTATCTCCATGACATGATTGGTAATTTCAAATCGGGTTGTAGCGGGTTTATCGACTGGAATCTGCTGCTGAACAGTGAGGGCGGGCCGAATCATCAGGGTAATCTGTGTGAAGCGCCCATTCAATACGATGCGCAAAACGACGTGCTGCGGCGTAACCACTCCTGGTATGGTATTGGCCACTTCTGCCGCTATGTGCGTCCGGGGGCGAGGGTCATGCTTTCTTCAAGTTACGATAATCTTCTGGAAGAGGTGGGATTTGTGAATCCCGACGGCGAGCGTGTGCTGGTGGTGTATAACCGCGATGTCCAGGAAAGGCGTTGCCGGGTGCTGGATGGCGATAAAGAGATCGCGTTAACGCTGCCGCCGTCAGGCGCCAGTACGTTGCTATGGCGGCAGGAGTCGATCTGA

***iolI1***

ATGATGAAGCTGGGATTTAATGAAGCGACCTGTATGCGAAACTCCACGCTGGCACAGGATGTTGTGTTGGCGGAACGTTTTGGCTATGACTACATCGAAATCCGTCTGGATATGTTGCAGGAGTGGTTGCAGAAACATACGTTAAGCGAGCTGGCAGACATTTTCGCCGTCGGCCACCTTAAGCCCTGGGGCTACAATTCGCTGGAAGACATTACGTTTTGTGATAGCGAAAGCTGGGCGGAGAAACTGCGGCAGTTGGCTTTCGCCTGCCATGCTGGCTCAGTGGTGGGGGGTGATTGTCTGGTTGTGGTGCCCACCATTCGTCAGGGGGGAAATTTTCCGCCAGGGGAAACGGTGAAAGATTCAGTGAAGCGTCTGCGCGAGATGGCGGCAGTGGCGGAAGAAAGCCGGATGCGGCTGGCCTTTGAGCCGATCGGTTCCGCAGGATGCTGTGTGCGCAGTCTTGCCATGGCGATGGAGATCGTAGATGCGGTCGATCGCAGTAATGTCGGGCTGGTCGTCGATGCCTTTAATCTTTACCTGCACGATCAATGGCGGGATCTGACCACGCTTCGCCAGATCCCTGTGGAAAAGATCTTCGTTTACCATATTGATGACGCCGATAATCTGCCGCTGGCAACGCTGGAGCATTGCCATCGTCTGTTTCCTGGCAACGGCGTGATCCCACTGCATGAGATCACGCATGAGCTAGTGCAGAAGGGCTATGAGGGGATCTGTTCGCTGGAACTGTTTAACCCTGGCTACTGGCAGATGGCGGCCAGCGAAGTCTTTGCGATAGGGGCGGAAAAGACTCGCCCCTTCTTAACAGCTTAA

TGTTGCCGCAGCAAGAGATGGACAACCGCCGCGCCCAGCAGCGCGCAGGCCGCGGCCAGAAGAAAGACCTGCTGATAGCCGATAAACGGCGTCAGCAGGCCCGCTATTGGTCCGGTAAATCCGTAGGCCAGATCCTGAAACGCTGACCAGACGCCGAGGGCCGTACCACGAATCTCCGGTGGCACTCTGCGCACAACCTCTACGCCCAGCGATGGGAACATCAGCGAACAGCCGCAGCCGGTAATCGCCGCGCCAATCAGCGCAGCGCCTGCTGACGGAGCGGCCCACATTACCGCCAGTCCGGTCCCCTCTACCAGTAGTGAAAAGGTTGCGACCGTGGCGCCGCCGTAACGGTCGGGAAATTTGGCGCAGAAAAAGCGGACAGCGATAAACGCAATGCCAAACAGCGTCATCGCAAAACCGGTGTTATCCCAGTGACGCTCGTTGAACCAGAGGGCGGTAAACGCGCTTAGGGTGGCAAAACCAATGCCCTGGAGCACAAGCCCTGTTCCGGGCCGCCACACCAGGCCGACTACCCGTAGCACTGGCACGCGGGGACGGGCTGTTGGGATATTCCCCGGAATGCCATAGATGACTCCGCTGGCGATGATCGGCAACAGCACGACGAGGAGCGCCGGAAGCGCCAGGCCGGCCCTGGCGTAAAGCGACAGCCCCAGCGGCGCGCCGATAGCCAGCGCACCGTAGGTCGCCATGCCGTTCCAGGAAATAACCTGACCCGCGTGGGTCGAGCCCGCCAGCCACATCCCCCAGGTTAAATTACCGGTCAAAATGAAACTTTCACCGATGCCCAGCAGCACACGACCAACAATCAGTATGGCCCATGCGAGCAGCGGAACCGGGTGAGCGATTAGACTGACCAGCATCAGCAGACCTGATGCGGCGCAGTAGAACTGACCGGTGATAACCGTCCGACGTCCGCCTGACGTGTCGGCTTTTCGACCCGCGGCGCCCCGGGTAAGCAAGGTGGCGATAAACTGGCTGCCTACCGCGATACCGATCAACAGATCGCTCAGATGCAACTGCTGCTGAACGTACAGCGGCAGCATGACTAACGGGATGCCAATAGTCAGATAGTTGAGAAACATCGCGGCGCTGATGCGAAACAGCGTGATCGATGTGGAATGTTGTGTGAGAGCTGGCAT STM4428

GGAATCGTCCTTGAACACGCCCCTTGTCCGATGCAGACACGGGGCGTGATGAGGCAGGCA

TTAACGTGCCGGGCGGTATTCGCGCCAGTAGTCGATAAGCATGCGGTATTTTTGTTTCACCGTTGCGATGAGCTGTTCATCGTTAAGCTGGCCGCCTAACCACTGGCGTGACGGTTCGCCAAAGATGGTCCGGCCGACGGCGAAACCTTTCACCCAGGGCATATCCGCTGCTGCGGCGAATCCTGACTTCAACTCGCTTTCCGGTGCGTCCAGGCCAAGTATCAATATACCGCGACATTCAGGGTCATAATGCTCAATATGCTGGCTGATTTCTCGCCAACGATCTGGGCTTAACGGCGGCAGTTTCCACCAGTCCGGCAGGATCCCCAGTTGATAAAAACGTTCCACAATCTGAGGATAGTATTGCTCATTTTGGTCTGCCGCATCGCGCGGCAAGATGACTTCCAGCAGCAGATCGTGACCAGACTGGCAGCAGGCGCGGTAAACCTCGTCAATGAGCGCCTCTTGTTCAAGACGTACCGACTCTGCATCGAGTGGATGATAGAAGACCAGACATTTCACGACGTGCTCTTGCGGCCAGCTGACAAGCTGAGAGCCAATATCGCCATGTTCCAGCTTCAGCGGATACGAACCGGGCATCTCAACCGGTCGGCCAATCCACCATCCCTGGCCGGTCACGTCGTTAAGCGCCTGCTGACCAAAGGTGGTATCGGCAAGGATGCCGCTATTATTCTGTAAACCGGCCTCAAGCGCCGCCTGACGTGCCCCTTCTAGCAGTAGCATCTTCAGGGGGGGGATCGCGGATTCACTGGCGCCGACTTCATTTGCGATATCCACCAG ***iolC2***

TTGCTTGCGATGGTCAAAGGCGAATACGCACAGCTCCGGCCAGTGCTGTTTACGGGTTGTGACCCGATGAAGGTGATTCAGCCGCGGGTCTTTGTCTGGCCGGGTGATGGATTGTTCACGCGCGAGGTAGTCGTCCAGCTCTTTTTTGGTTGGCATGGCGGGGGCACAACCGTGGCGCGATACCACTAACGCGCCGCAGGCGTTGCATAACGGCAGGCTTGCTCCCAGCTTTCATCGTTCAGGTATCCCCGCAGCAGTCCTGACATAAAGGCATCGCCCGCGCCCAGAACGTTCAGAACGTCTACGCGAACGCCGCTATGTATCTTCACCTGCGACCAGTCATCGGCGATGTTGCCTTCAAAGACTGAGCAGCCCAGCGCGCCGCGTTTGCACACCAGTACGGCCTGGGTCAACTGACGGACGCGCCGCAGGGCGGTCAGCGTGTCGGTACTGCCGCCGGCGATATGGAATTCTTCTTCGGTCCCAACGATCAGATCGAAATGGCGCAATACCTGCTGGAGCTGTTCGGTAACCTGTGATGAGGCGATGAAACGCGTTTCACCGTCTCCTAGCGACGTCAGGCCCCACAGTACGGGGCGGTAGTCGATATCCAGTGCCCGCCGCAGCCCGTGTTTTTGCGCATACTCCAGCGCTTTAAGAACGGCGGCGCGCGTATTGGGGTGAGAAAGATGGGTACCGGTGACGGCTAATGCGCGTGAAGAAGCGATGTAATCTTCGCGGATATCCTCAGGTGTTAGCGCCATGTCCGCGCAGTTATCGCGGTAGAAGATCAGAGGAAAGGTGTCCTGATCTTTAATGCCGAGGATCACCAGCGCGGTCAGACGATCGCGATCCGACAGCAGGCACTGCGTATCCACACCAACGCGGTTTAATTCTTCACGAACGAAACGTCCCATATGCTCATCGCCGACGCGGGCCAGCATAGCGGAACGTAATCCCTGAATGGCCGTGCCGTAGGCAACATTGCCTGACGATCCCCCTAAATATTTAGCGAAAGTCGTCATATCCTCCAGCCGTGACCCGATTTGTTGACCATACAAATCAACGGCGACTCGTCCCAGACAGATAACGTCCAGCGGCTTTTGCTCACTACTGACTGCTTTATTCATTGACAT ***iolC1***

ATCCCCTAACAGAAGACGGTGTTATTTTTGCTCATACTAAGGTTTGGTGATTTCATTTTCAATAAAAATGGAAATAATGTTTTCATTTATTGTTTGAACAAGATCACAGAAATGGCATTTCCGGGCAACGGGCATGATCGTTTTTTGTTGTGTTTTTTGTTTTAATTGATTGATTATAAATGTGTTATTTATTTTAAAATCGCATGGAAGATAAATTTCATTTTCATGAAAAATACGCCTGAATGTCGAAATTTTTTAACCGTTTTTTGATCTCCTTCCCATAAATGAAATATAAATTCCATATAATTGTGAGAACGGAAATTTTGATCCTATGATAAGTCTCTGTGTTTTTGGGGCTGAAGCGATGCATAAGCAGGCAACGACAGCCGAACGGTTGATAAAAGCAGGTGGGGTAC

***iolD1***

ATGAAAACAATCAGGTTGACCATGGCGCAGGCTTTGGTGCGCTTTCTTGATAATCAGTACATCGACGTAGACGGCAGCGAAATCAAATTTGTAAAAGGGATTTTCGCCATTTTTGGCCACGGGAACGTCGTCGGATTGGGGCAAGCGCTGGAAGAGGACTGTGGCCAACTTAGCGTTCATCAAGGGCGTAACGAACAGGGAATGGCGCATATCGCGACGGGATTTGCCCGCCAGATGCGTCGCCATCAGATTTATGCCTGCACCTCCTCAGTGGGGCCAGGGGCCGCCAATATGATCACCGCAGCGGCGACCGCGACGGCTAACCGTATTCCACTGCTTTTGCTGCCGGGCGATGTGTACGCATCGCGTCAACCCGACCCGGTTTTGCAACAGGTTGAACAAGAACACGATTTGACGCTGAGCACCAATGACGCTTTCCGTGCAGTTAGCCGCTACTGGGATCGCATTACGCGTCCGGAACAGCTAATGAGCGCCTGTATCAGCGCGATGCGGGTGTTAACCGATCCGGCGGATACGGGGGCCGTGACGCTTTGCCTGCCACAGGATGTGCAGGGTGAAGCCTGGGATTATCCGGATTATTTTTTCGCTCGCCGGGTCTATCGTCTTGAGCGTCACGCGCCGACGGAGCCGATGCTGAACGAGGCGGTTGCGCTGATTCGCCGCCACCAGCGGCCGCTGATCGTTTGCGGCGGGGGCGTGAAGTACTCGCAGGCTGAAGAGGCGCTGCTGAGATTTGCCGAACGCTGTCATCTGCCGATTGCTGAAACCCAGGCTGGCAAGGGAGCGCTCAGTTCTGCACACCCGCTGAACGTCGGCGGGATTGGCGAAACCGGTTCACTGGCGGCGAATCTGCTGGCGCAGGAGGCCGATCTGATTATCGGTGTAGGGACGCGCTATACCGATTTCACCACCTCCTCAAAGTGGATCTTCCAGAATCCCGACGTGCGCTACTTAAATATCAACGTTAGCCGCTTTGATGTCTTCAAGCTGGATGGCGTACAGATGCAGGGTGACGCTCGCGTCGCCTGA

CGCAGCTTAGCGAACGGCTGGCCCAGGAGCATTATGCTTCGCAATGGGGTGAGACTATTCACCGCGTCCGCTCGCAATAT

***iolD2***

ATGGCGGAAGTTGAGCGCGTCTATGCTGTGGAATATAGCGGAGAGGGCTTCAAACCTGAAATTGAGGATCATATGGATACTCAAAAGGTGTTTGAAGAGTTTAATGAGATTACGCGGTCGTGGCTGACCCAGACGCGCGTGTTGGGTGTGCTTAACCGGATGTTGCCGGAAAACGCGCTGGTGGTGGCGGCGGCGGGCAGCCTGCCGGGCGACCTCCAGCGTGTCTGGCAAAGCCGCGGCGAGAATGATTACCACGTCGAGTACGGCTACTCCTGTATGGGCTACGAAGTCAATGCCGCATTGGGGGCCAAACTGGCGCAGCCGGAGCGCGAGGTGTACAGCTTCGTGGGCGACGGTTCGTTCATGATGCTGCACTCTGAGTTGGTCACTTCCGTCCAGATGGGGAAAAAGATTACCGTCATTTTGCTCGATAACATGACCAACGGCTGTATCAATAATCTGCAAATGGAACACGGTATGGACAGTTACTTCACCGAGTTTCGTTTCCATCAGCAGGAGAGCGGTCGTCAGGAAGGCGGGTTTATCCCGGTCGATTTCGCTCGCATCGCTGAAGGATATGGTTGTAAAAGCTATCGCGTCACCACCATTGAACAACTGCATGAAGCGTTGGAAGATGCTCGTAAACAGACCGTGAGCACGCTGATAGACATAAAAGTGCTTCCCAAAACGATGGTGCATAAGTACCTGAGCTGGTGGCGCGTTGGTGGGGCGCAGGTATCCCGTAGCGAACGTATCCAGGCGGTAGCGCGTATGCTTGAAGAACATATCGGACAGGCCCGGCAGTACTGATTAATTTGCCGTCGTTCAGGTTGCAGAAGTGTGGTTATTGTTCTGCAACCTGAACGATACAGCGTCAACGCATTGTTTGCTTTTCAGAACGGCAAAATAAAACGATCCGTTTTTCGCAGAACTACGGATGCTTTTCTTTTGCGGTTTTAAATTTACTATTCATGAGATTAAGAGGTACGA

***iolG2***

ATGAAAAAGCTTCGATGTGGTGTTATTGGCCTCGGTCGGGTAGGTAAAATGCATGTTGAGAATATGTATTTACTGCCTCAATTAGATATTATCTGCGCCGCCGATTATTTTATTGAGGAGATGAGCGATTGGCTTTATTCGGTAAATATAACTTCAGGGTATAAAAATTATCAGGAACTTTTACAGCGTGATGATATAGAGGCAGTTTTTATTTTCACCTCAACGGATATGCACGAAGAGATCGTCACGGCGGCAGCGCAGGCGGGGAAGCACATTTTTTGTGAAAAACCGCTCAGCATGAATGAGGATGAGCAGGCCTCAATGGCGGTGCTGAGAAAAGTGAAAGAGAAGGGGGTGACGCTGCAGGTTGCCTTTAATCGCCGTTTTGATCCTCAGTTTCACGAGGTCTTTGAGCTGGTGCGCAGCGGCAAGATTGGTCGGCCCCAGATGATAAAAATCACCTCCCGCGATCCGGACCTGCTACCCCATGATCTGATCAAACGTATTGGCGGTCTGATTTTTGACTTCACCATGCATGACTTTGATATGGCTCGTTTTATGATGCAGGATGAAGTCAGCGAAGTCTATGTTAAGGGCAATACGCTGATTGATCCCAGCCTGAAAAATATTGATGATGTTGATACGCTTGCGGTGATGCTGACTTTCAGAAACGGCGGCTACGCGCTCATTGATAACAGCCGTCGGGCGGTATATGGCTATGACCAACGGGTGGAGGTTTTTGGCTCAGAAGGGATGGCGTATGCGGATAACGTTAGTGAGTCGACGGTGAAAGTTTTCAACAGCCAGCACTGCATAATGAAAAATCCCCTTCCTGATTTCACGGTCCGTTATCGCGAAGCTTACCGAACGGAAATATTACACTTTATTGATTCAGTCCTGCATCATACGCCGGTTGTCTGTACCGGCGAAGACGCATTATTGGCCCAGCGTATCGCTATTGCCGCGCAGCAATCATTAAAAAGCGGGCTGCCGGTGAAAATAACAAGCGATATTTATCTTTAA

CTGCATCTATTAATAATGAAGAAACTCTCGCACTGAAAAGTGTGAGAGCTTTTCTATACCGAGGATAATAAA

**STM4434**

ATGAAAGAAATGCAGGCAACGCTGCCCCAGACATTTTATATAAAACCAGGTAAGTTTATCTGGAGCTACCTGGGAACATTTTTATTTATGTTGGGCGGCTGTATTGAGAACAGCTGGTTATCTGCCTGGTTAAATACGCAGGGGTTTGATCAGGCGCATATCGGGCAGATTTTTGCCGGGTATGGGATTGTGGTCGCGATTACCTCCTGGCTATCCGGCGTCTGTGTGGACGTTTTTGGTCCTAAAAAGGTGATGGTTACCGGTTTTATCGTCTATCTTCTGGCTTCCGTCGCGTTTCTGAACTTTGCGCTACCCAGCCATGATTTTGGCGCAATTCTTGTTACCTATATGCTGCGTGGCGTGGGGTATCCGCTGGTCTGTTACTCCTTCCTGGTACGCCTGACCATCCAGTTGGATAATCACCAGCAAGGGATCGGCACCTCGCTATTTTGGGTGGTCTATAACCTTGGTTTTACCATCATTGGGCCGGTGGTGGCGGCGTCGTTAATTCCTGAGTTGGGGCATATCAACGTGATGTGGGCCGGGATGGGCGTTGCGCTGCTGGGGGTTCTCTTCATGCTGGTGCTGGAACGCAATGAATTCATTCTGAAACCGCGAACGACGCCGGTCTTCAAAGAGTTATCTGCGGGTATCTCCATTATGTATGAACGGCCACGGATCGGTCTGGCGGTGATTGTCAAAACTATTAACGGTCTTGGCACCTATGGTTTTGTTGTCGTATTACCTCTGTTTCTGCTGGATAAGCACTTTACGCTTGAGGAATGGGCGAGTATTTGGGGGATTACCTTTATCTCTAACCAGGTCTTCAACATTATCTTTGGCTGGATGGGCGATAAAATTGGTTTTCGCCGAACCATCCAGATTTTTGGCAGCATCCTGACCGGTGTCGCTACGCTGATTGTTTACTGGGTGCCGATGATATGGGGGCATAACTATGTCGCCTTTATGCTGGCGATGTGTTTGTGGGGAGCCGGGCTGGCTGGCTTTGTGCCAATGACGCCGCTGGTGCCGATGATGGCGCCGGATAAGAAGGGGGCGGCGAACTCGGCCGTGAACTTTGGCTCCGGCCTGGGGAATTTCGTTGGGCCAGCGCTGGTCTCCGTTCTGGCAGGGTTCGGCACCGGCGTGGTGATGTATACTATGGCGGGGCTCTATTTATTCAGCGGCATCCTGGTGCAGTTTCTTAAAGTGCCTGGCGAGAAATAG

CTTCAAATATTCTTCAAAAATCCCTTATAGCCTGTGATGTTCATCACTAAATTAAACCGGTTTTTATCAAGATTGATAAAGCCGGTTTTTTCACCCTACGTTAAATTGTACTAACGGATTACGGTTATTTACCCACTCATATACGTTCAGGGAGAAAATA

***iolI2***

ATGAATATCGAAAAAACACGGTTTTGTATTAACCGGAAAATTGCACCGGGTCTAAGTATAGAAGCATTCTTTCGACTGGTTAAACGTCTTGAGTTTAATAAAGTGGAATTGCGTAATGATATGCCAAGCGGCAGCGTTACTGATGACTTGAATTATAATCAGGTGCGTAACCTGGCAGAAAAGTATGGACTTGAAATTGTCACAATTAATGCGGTCTATCCCTTTAACCAACTGACCGAGGAGGTAGTAAAAAAAACGGAAGGATTATTACGAGACGCACAGGGAGTCGGCGCGCGGGCATTAGTTCTTTGTCCGCTGAATGATGGAACGATTGTGCCGCCGGAGGTTACGGTTGAGGCAATAAAACGACTGAGCGATCTCTTTGCCCGGTATGATATTCAGGGGCTGGTTGAACCTTTGGGATTCCGCGTAAGTTCGCTGCGTTCTGCCGTCTGGGCTCAGCAACTGATCCGGGAAGCAGGTTCCCCCTTTAAGGTTTTGCTGGATACCTTCCATCATCATCTGTACGAAGAGGCCGAAAAGGAGTTTGCGTCGCGCATTGATATTTCTGCTATCGGGCTGGTGCATTTGTCCGGTGTGGAAGACACCCGACCAACGGAAGCATTGGCTGATGAGCAGCGTATTATGCTGAGTGAAAAGGATGTAATGCAGAATTACCAGCAGGTGCAGCGTCTGGAAAATATGGGATATCGCGGTATTTACGCTTTTGAACCTTTTTCTTCCCAGTTGGCTTCCTGGAGTGAGGCGGAAATAGAGGAACAAATTAATCGCAGCGTGTCATTGCTGCTGCAATGA

TACAGCTAATTTCCAGGTGACCGTTTTCAGTTTCCGCAGCGTGGTATTTACA

***iolH***

ATGAAAATTGCTTTTGATGTCGATGTTATTAAACATTTACCCATTACCCAAATGGTACGGCAGGTTTCCGAGTGGGGGTATAAATATATTGAGCAGTCGCCGCACCCGCGTATTAATCCGTTTTATAAACACCCTAAAGCTGGTCGCGATACCATGCAGGAATATAAACGCGCCTTGCAGAACTACGGTGTGGAAATCTCGTCATTTATTGTGGTTTATCGTTGGTCCGGCCCCGATGAAGAGCGTCGGCGCGCCGCGGTAACCAACTGGAAGCGGATGATTGAGATTGCCGTTGATATGGGCGTACAGGTGATTAATACCGAGCTTTCCGGGACGCCGGATGAGCCGGAGATTTGTGAAGAGATGTGGTATCGCTCCATGGAGGAATTGCTGCCGATCGTGGAACGAGAGGGAATTCGAATCGAAATTCAGTCTCACCCCTGGGATTTCTGCGAACTGAACGATGAGACGGTCGACATGGTGCAGTCTTTGCGTAGTGATAACGTGACCTATCTGTACAGCGCACCGCATGGCTTCTTTTACGATAAAGGGCAGGGCGACGTCGCAAGGATGCTGAACTATGCCGGGGCCGATCTTTCGCATGTTCTGCTGGCCGATACGCACAACCACACGCTTCCCTGCCGCTATATCATGAACCCGCCTGGCGTGAATGCGACGATTCATCAGCACATCGGGCTGGGAGAAGGTGAGGTCGATTTTGACGCGCTATTCCAGGCACTGCGTGAAATGGATTTTGCAAACCGCACGTTTAAGGTGGGCGGTGAGGCAATTATCACCACGTCGCTGTTTGGTTATCCTGAGAAAATGAGTGTTCAGGCGGTGGAAACGCGCGAGCGGATCGAGCGGGAGCTACTCGGACGTTAA

AAACGGGTGGATTTGCCTGTACCGCGCCACAGGCTGGCGCGGTCATTTGCGGCAATTACCCTTCGTTTTCCGCCAGCTCGCGCAGATACTGGAAGATCTGACGCGCTGATTTCGGCGGTTTGTTGCCTTCTTTCTCTTTCTTCGCATTGCGGATCAATGAACGAAGCTGTTGACGATCGGCGTGCGGCCACAGGGTCAGTACTTCGGCTACCGCGTCATCGCCTTCGACGATCAGACGATCGCGCAGATGCTCAAGTTTGTGAAACAGCACCACCTGCTGATTATGGCGGTTCTTCAGCTTGTCCAGCGCCTGGCGAATAGGTTCAACGTCGCGTTGGCGCAGCATTTTTCCGATAAGCTGCAACTGGCGGCGGCGTCCTTCCATTTTGATACGCTGGGCCAGTTC
