# Supplementary material for: Hierarchic regulation of a metabolic pathway: H-NS, CRP, and SsrB control myo-inositol utilization by Salmonella enterica
Source: Microbiol Spectr. 2023 Dec 14;12(1):e02724-23. doi: 10.1128/spectrum.02724-23 (PMC10783015; doi:10.1128/spectrum.02724-23)
Supplement: Table S2 — Oligonucleotides used in this study. [file spectrum.02724-23-s0004.docx]

Supplementary Table S2: Oligonucleotides used in this study.

| target | sequence 5´-3´ | Length [bp] |
| --- | --- | --- |
| *EMSA* | | |
| P*_iolR_* | TCGCCGGGAGACGTTAAAG/  CGACTGCGGATATCATCCTG | 469 |
| P*_iolR_* (CRP) | GGAATTCACGAAAAGAGCCAGTTCG/  CGGATCCGTTTAGACATGCATGATAC | 299 |
| P*_iolT1_* | CGAATTCATCAATAAAATCAAGTAACTTC/CCGCCCAGTGCGGC | 362 |
| P*_iolT2_* | GGAATTCTCTCGTTTCACAACCTATG/ CGGATCCTCTGAGACATAATCCCTCCC | 347 |
| P*_iolA_* | CCATTGATGACACACC/  CCGGATTAGTCACCGG | 334 |
| P*_iolE_* | GTTGGCTTTAGCAACC/  GGCTCCCACTTAATGAAACG | 385 |
| P*_reiD_* | CTTCAGCCTAAAACGCATAC/  TGAGCCTTATTCTCTCGTTC | 364 |
| P*_srfJ_* | CCGGAATTCTCTTCGATCGTCTGAACGC/  GCCGGTACCAGGGAAGTTCCGGATAAAAG | 252 |
| P*_iolC1_* | GAAAATGAAATTTATCTTCCATGCG/  ATAACGTCCAGCGGCTTTTG | 275 |
| P*_iolC1.2_* | TCGACATTCAGGCG/  CATTTCCGGGCAACG | 134 |
| P*_iolD1_* | ATACGCCTGAATGTCGAAAT / GATTATCAAGAAAGCGCACC | 430 |
| P*_rssR_* | GCAgagctcTGGCCGTATGCAGCG/ CGGggtaccTTATCGCGGGTAGTC | 499 |
| *reiD* (fragment I) | CGTGTCCGGGTCTACGTG/ AGCCGAATTGGCTCATAG | 292 |
| *reiD* (fragment II) | CTATGAGCCAATTCGGCT/  GCGACCATTCTCATGACG | 346 |
| P*_iolE_* (fragment III) | ATCGCAAGGACTATCGTAAAGC/ AAAACCGGTGAGCGCGGCATC | 450 |
| *iolE* (fragment IV) | GCCGCGCTCACCGGTTTTTCC/ AAGCTGCCATTCGCTGTCGC | 300 |
| *iolE* (fragment V) | GACTCATGGAGAATACCGATC/ TCGATCGCATACTTATCCAG | 556 |
| *iolG2* (fragment VI) | GCTCGTAAACAGACCGTGAG/ TCCTGATAATTTTTATACCC | 530 |
| *iolSTM4434* (fragment VII) | AGTTTTCAACAGCCAGCACTGC/  ACATAAATAAAAATGTTCCC | 395 |
| *iolI2* (fragment VIII) | TTTGTGCCAATGACGCCGCTG/ CGCATTAATTGTGACAATTTC | 595 |
| *iolI2* (fragment IX) | GTCTATCCCTTTAACCAACTG/  GAAGGTATCCAGCAAAACC | 300 |
| *iolH* (fragment X) | TCGCGCATTGATATTTCTGC/ TATCAACGGCAATCTCAATC | 614 |
| P*_iolG2_* | GGAATTCAAGTACCTGAGCTGGTGG/ CGGATCCGCTTTTTCATTCGTACCTCT | 301 |
| P*_iolI2_* | ATTTCGTTGGGCCAGCG/ CGATATTCATTATTTTCTCC | 301 |
| P*_argS_* | CAACCTTTGATTTGATTGG/ AAGAGCCTGAATATTCAC | 195 |
| *SPR spectroscopy* | | |
| P*_iolR_* | TTGCTCAGAACCCAGC/ CTGCGGATATCATCCTGC | 188 |
| P*_iolT1_* | AACGATAAAAAACGCCAG/ CTTATGTCGGTCATTGCC | 161 |
| P*_iolT2_* | AATTTGTCAGGGGCCG/ AATCCCTCCCTTGCCTG | 146 |
| P*_iolA_* | CTGCTGCTCAGTGTCG/ GCTGCACATACCCTCG | 241 |
| P*_reiD_* | GGTGTGTCATCAATGG/ TGTGGTGCCGAAACCC | 126 |
| P*_iolC1_* | ATCATGCCCGTTGCC/ TCCAGCGGCTTTTGC | 182 |
| P*_iolD1_* | ATGCATCGCTTCAGCC/ ACGCCTGAATGTCG | 136 |
| P*_iolG2_* | AAGTACCTGAGCTGGTGG/ GCTTTTTCATTCGTACCTCTTAA | 301 |
| P*_iolI2_* | ATTTCGTTGGGCCAGCG/ CGATATTCATTATTTTCTCCCTGAA | 301 |
| P*_iolE_* | GGTCAATATCGCAAGGACTATC/ CTGGCTCCCACTTAATGAAAC | 324 |
| P*_argS_* | CAACCTTTGATTTGATTGG/ AAGAGCCTGAATATTCAC | 195 |
| *cloning* | | |
| *hns* (in pBAD/HisA) | GACGAGCTCATGAGCGAAGCACTTAAA/  GGAATTCCTTATTCCTTGATCAGGAA |  |
| *crp* (in pBAD/HisA) | GCAGAGCTCATGGTGCTTGGCAAACCGC/ GGAATTCCTTAACGGGTGCCGTAGACGAC |  |
| *P_iolA_* (in pUTs-*lux*) | GCAGAGCTCCATATGCATGAACCG/  CGGGGTACCCTTAAACCGATGTTG | 500 |
| *srfJ* (in pUTs-*lux*) | GCAGAGCTCCCCAGTGCCTGCCGG/  CGGGGTACCCAGATCGACTCCTGCC | 500 |
| P*_srfJ_* (in pDEW201) | CCGGAATTCTCTTCGATCGTCTGAACGC/  GCCGGTACCAGGGAAGTTCCGGATAAAAG | 252 |
